# Exploring hypoxia-related genes in spinal cord injury: a pathway to new therapeutic targets

**DOI:** 10.3389/fnmol.2025.1565430

**Published:** 2025-05-20

**Authors:** Shihuan Cheng, Le Li, Mengmeng Xu, Ningyi Ma, Yinhua Zheng

**Affiliations:** ^1^Department of Rehabilitation Medicine, The First Hospital of Jilin University, Changchun, China; ^2^Department of Rehabilitation Medicine, China-Japan Union Hospital of Jilin University, Changchun, China

**Keywords:** spinal cord injury, hypoxia-related differentially expressed genes, diagnostic model, functional enrichment analysis, gene regulatory networks

## Abstract

**Introduction:**

Spinal cord injury (SCI) remains a debilitating condition with limited therapeutic options. Exploring hypoxia-related genes in SCI may reveal potential therapeutic targets and improve our understanding of its pathogenesis.

**Methods:**

We developed a diagnostic model using LASSO regression and Random Forest algorithms to investigate hypoxia-related genes in SCI. The model identified critical biomarkers by analyzing differentially expressed genes (DEGs) and hypoxia-related DEGs (HRDEGs). Gene Ontology (GO), Kyoto Encyclopedia of Genes and Genomes (KEGG), Gene Set Enrichment Analysis (GSEA), and Gene Set Variation Analysis (GSVA) were conducted to explore the biological roles of HRDEGs. The model’s accuracy was validated using receiver operating characteristic curves, calibration plots, decision curves, and qPCR experiments.

**Results:**

The diagnostic model identified Casp6, Pkm, Cxcr4, and Hexa as critical biomarkers among 186 HRDEGs out of 9,732 altered genes in SCI. These biomarkers were significantly associated with SCI pathogenesis. GO and KEGG analyses highlighted their roles in hypoxia responses, particularly through the hypoxia-inducible factor 1 pathway. The model demonstrated high accuracy, with an area under the curve exceeding 0.9. GSEA and GSVA revealed distinct pathways in low- and high-risk SCI groups, suggesting potential clinical stratification strategies.

**Discussion:**

This study constructed a diagnostic model that confirmed *Casp6*, *Pkm*, *Cxcr4*, and *Hexa* as important biomarkers for SCI. The findings provide valuable insights into SCI pathogenesis and pave the way for novel treatment strategies. The integration of multi-omics data and comprehensive bioinformatics analyses offers a robust framework for identifying therapeutic targets and improving patient outcomes.

## 1 Introduction

Spinal cord injury (SCI) is a severely disabling neurological condition with far-reaching and complex impacts. Approximately 270,000 patients with SCI across North America, with over 12,000 new cases, are reported annually in the United States (Sterner and Sterner, 2022). SCI results in lifelong physical disability and a significant decrease in quality of life and imposes a substantial economic burden on society. Patients with SCI often require long-term rehabilitation, which is accompanied by high medical expenses, continuous care needs, and significant financial burden due to loss of work capacity (Yari et al., 2024). Although current treatment methods, such as surgery, drug therapy, and rehabilitation training, play a fundamental role in SCI treatment, their effectiveness in promoting neurological recovery is still limited. Unresolved limitations include high treatment costs, unstable outcomes, and significant individual patient differences (Stanciu et al., 2023). Therefore, an in-depth exploration of SCI pathogenesis and an active search for new treatment targets and biomarkers are paramount for improving patient prognosis and reducing the socioeconomic burden.

Hypoxia is a key factor in the pathophysiological process following SCI, significantly affecting nerve cell survival, inflammatory responses, and tissue repair (David et al., 2021). Previous studies have shown that transcription factors (e.g., HIF-1) can be activated under hypoxic conditions, which regulates downstream genes to promote angiogenesis and erythropoiesis, ultimately improving blood supply and tissue oxygenation (Huang et al., 2022; Zhang et al., 2023). However, despite its recognized importance, the precise mechanisms by which hypoxia influences SCI pathogenesis, interacts with hypoxia-related differentially expressed genes (HRDEGs), and affects the onset and progression of SCI remain unclear. A deeper understanding of these hypoxic mechanisms may provide critical targets for developing novel therapeutic strategies for SCI.

Recently, studies have focused on gene expression changes in SCI through bioinformatics approaches; however, few have comprehensively integrated multi-omics data, constructed diagnostic models, or explored the relationships between gene regulatory networks and immune infiltration (Shang et al., 2024). This study addresses these gaps by systematically integrating two independent datasets to identify 186 HRDEGs and constructing an efficient diagnostic model for SCI. The model highlights the pivotal role of key genes such as Casp 6, Pkm, Cxcr 4, and Hexa in SCI, offering valuable insights into SCI pathogenesis and developing new therapeutic approaches.

## 2 Methods

### 2.1 Data source

The R package GEOquery (version 2.70.0) was used to retrieve SCI datasets GSE5296 and GSE47681 from the Gene Expression Omnibus (GEO) database.^1^ The samples in these datasets were derived from *Mus musculus* (Supplementary Table S1). Both datasets used GPL1261 as the chip platform. Dataset GSE5296 included six SCI and four control samples, whereas dataset GSE47681 included eight SCI and four control samples (Wu et al., 2013; Clough and Barrett, 2016). All available SCI and control samples from these datasets were included for analysis.

Hypoxia-related genes (HRGs) were obtained from the GeneCards database,^2^ which offers extensive information on human genes. The keyword “Hypoxia” was used for the search, and only HRGs classified as “Protein Coding” with a “Relevance Score” of above five were retained, yielding a total of 123 HRGs. A set of HRGs was obtained from PubMed^3^ using “Hypoxia” as the keyword, yielding 200 HRGs. After merging these datasets, removing duplicates, and converting them to mouse-derived genes, 285 HRGs were identified (Supplementary Table S2).

The R package sva (version 3.50.0) was used for batch processing on datasets GSE5296 and GSE47681 to obtain the integrated GEO datasets (Combined Datasets) containing 14 SCI and eight control samples. The Combined Datasets were processed using the R package limma (version 3.58.1) for standardization, probe annotation, and normalization. Principal Component Analysis (PCA) was used to reduce dimensionality by extracting and transforming feature vectors (components) from high-dimensional data into a low-dimensional format, visualized in 2D or 3D graphs. PCA was conducted on the expression matrix before and after removing batch effect correction (Ben Salem and Ben Abdelaziz, 2021) to validate the removal of batch effects.

### 2.2 SCI-associated HRDEGs

The samples from the Combined Datasets were divided into two groups: SCI and control. Differential gene expression analysis between the SCI and control groups was performed using the R package limma (version 3.58.1). A |logFC| > 0 and *p* < 0.05 threshold was applied to identify differentially expressed genes (DEGs). Genes with logFC > 0 and *p* < 0.05 were classified as upregulated DEGs, whereas those with logFC < 0 and *p* < 0.05 were defined as downregulated DEGs. A volcano plot was generated to visualize the differential analysis results using the R package ggplot2 (version 3.4.4). The Benjamini-Hochberg (BH) method was used for all *p*-value corrections.

To identify HRDEGs associated with SCI, all DEGs with |logFC| > 0 and *p* < 0.05 were intersected with HRGs. A Venn diagram was used to identify the HRDEGs. A heat map of the top 20 HRDEGs was plotted using the R package pheatmap (version 1.0.12).

### 2.3 Gene ontology (GO) and Kyoto encyclopedia of genes and genomes (KEGG) pathway enrichment analyses

GO analysis is a widely used technique for conducting large-scale functional enrichment studies, including Biological Process (BP), Cell Component (CC), and Molecular Function (MF) (Mi et al., 2019). KEGG is a well-established database containing extensive information on genomes, diseases, drugs, and biological pathways (Kanehisa and Goto, 2000). The R package clusterProfiler (version 4.10.0) was used for GO and KEGG pathway enrichment analyses of HRDEGs. The criteria for entry screening were *p* < 0.05 and a false discovery rate (FDR) value (*q*-value) of < 0.05.

### 2.4 Gene set enrichment analysis (GSEA) and gene set variation analysis (GSVA)

To determine the contribution of genes to the phenotype, GSEA (Subramanian et al., 2005) was used to analyze their distribution trends within predefined gene sets based on their ranking by correlation with the phenotype. For this analysis, genes from the Combined Datasets were first sorted according to their logFC values between the SCI and control groups. The R package clusterProfiler (version 4.10.0) was then used to perform GSEA on all genes in the Combined Datasets. The following parameters were applied in GSEA: seed, 2020; minimum number of genes in each gene set, 10; maximum number of genes, 500. The gene set m2.all.v2023.2.Mm.symbols, obtained from the Molecular Signatures DatabSCIe Database, was used for the analysis. The screening criteria for GSEA were *p* < 0.05 and FDR < 0.05.

GSVA (Hänzelmann et al., 2013) is an unsupervised, non-parametric method to evaluate chip nuclear transcriptome performance. It converts gene expression data from different samples into an expression matrix of gene sets between samples, allowing evaluation of pathway enrichment across different samples. The m2.all.v2023.2.Mm.symbols.gmt gene set was obtained from the Molecular Signatures Database (Liberzon et al., 2011). The R package GSVA (version 1.50.0) was used to perform GSVA on all genes in the Combined Datasets. The functional enrichment difference between the SCI and control groups was calculated. The screening criterion for GSVA was *p* < 0.05.

### 2.5 Construction of a diagnosis model for SCI

Logistic regression analysis was performed on HRDEGs to construct a diagnostic model for SCI. The association between the independent variables (HRDEGs) and the dependent variables (SCI group vs. control group) was analyzed. HRDEGs with *p* < 0.05 were selected to build the logistic regression model. Next, Least Absolute Shrinkage and Selection Operator (LASSO) regression was performed on the HRDEGs included in the logistic regression model using the R package glmnet, with the following parameters: set.seed(500) and family = “binomial.” A penalty term (absolute value of slope × lambda) was added to the linear regression model to mitigate overfitting and enhance the model’s generalization ability. The LASSO regression analysis results were visualized through diagnostic model plots and variable trajectory diagrams. The HRDEGs included in this model are referred to as model genes. Finally, the LASSO risk score was calculated based on the risk coefficients from the LASSO regression analysis using the following formula:


$$\text{RiskScore}=\sum_{i}\text{Coefficient}\;({\text{gene}}_{i})*\text{mRNA Expression}\;({\text{gene}}_{i})$$

Random Forest (RF) (Gregory et al., 2022) is an ensemble learning method that integrates several decision trees via bagging (bootstrap aggregation). In this approach, multiple decision trees are created, and when making a prediction, the results from all trees are aggregated through majority voting to reach the final outcome. To build a model using RF, the random Forest package was employed based on the expression of HRDEGs included in the regression model within the expression matrix of the Combined Datasets. The seed was set to 234, and the number of decision trees was set to 200. Mean Decrease Gini (MeanDecreSCIeGini) was used to assess the average decrease in the Gini coefficient, which measures node impurity. A higher Gini coefficient indicates lower purity and greater impurities. Therefore, MeanDecreSCIeGini reflects the average reduction in impurity at variable separation nodes across all trees, with a larger value (v) indicating greater importance of the variable. Next, ten-fold cross-validation was performed five times and combined with the cross-validation curve to determine the number of variables. Cross-validation ensures the robustness of the model by using different training and validation sets, avoiding biased results and insufficient training data. The training set was used for cross-validation, retaining several variables with relatively low error rates. Important variables for subsequent analysis were selected based on their MeanDecreSCIeGini values. Genes identified from the intersection of HRDEGs screened by LASSO regression and RF analysis were chosen as key genes (mRNA) for further investigation.

### 2.6 Validation of a diagnostic model for SCI

The R package rms was used to develop a nomogram for the key genes. A nomogram employs a cluster of disjoint line segments to visually represent various independent variables within a rectangular coordinate system. Based on multivariate regression analysis, the nomogram translates the risk of each variable into a cumulative total risk score, which can then be used to predict the probability of an occurring event. A calibration curve was plotted to assess the predictive accuracy of the model. This curve evaluates the model’s performance by comparing the actual probabilities with those predicted by the model under different circumstances. It is primarily used to determine how well the logistic regression fits the actual data.

Decision curve analysis (DCA) offers a simple approach to assess molecular markers, diagnostic tests, and clinical prediction models. The R package ggDCA was used to generate a DCA plot, which evaluates the accuracy and resolution of the logistic regression model. In addition, the R package pROC was used to draw the receiver operating characteristic (ROC) curve for the Combined Datasets. The area under the ROC curve (AUC) was calculated to measure the diagnostic effectiveness of the logistic regression model. An AUC between 0.5 and 1 indicates diagnostic accuracy, with values closer to 1 representing greater diagnostic performance.

### 2.7 Protein–protein interaction (PPI) network

PPI networks consist of proteins that interact with each other. This study used the STRING database (Szklarczyk et al., 2019) to search for interactions between predicted and known proteins. The biological species was set to human, with a confidence level of ≥ 0.150, to construct a PPI network for key genes. Cytoscape software was used to visualize the results. The GeneMANIA website (Franz et al., 2018) was used to predict functionally similar genes to the selected key genes and construct an interaction network.

### 2.8 Construction of regulatory networks

Transcription Factors (TFs) regulate gene expression by interacting with HRDEGs at the post-transcriptional level. The regulatory effects of TFs on HRDEGs were analyzed using TFs retrieved from the ChIPBase database.^4^ The mRNA-TF regulatory network was visualized using Cytoscape (Shannon et al., 2003).

MicroRNAs (miRNAs) have a crucial regulatory function in biological development and evolution, with the ability to regulate multiple target genes. Conversely, a single target gene can also be mediated by various miRNAs. To analyze the relationships between HRDEGs and miRNA, related miRNAs were obtained from the TarBase database^5^ and the StarBase v3.0 database.^6^ The mRNA-miRNA regulatory network was visualized using Cytoscape.

RNA-binding proteins (RBPs) regulate genes and BP, such as RNA modification, alternative splicing, transport, synthesis, and translation (Singh, 2021). The target RBPs of HRDEGs were predicted using the StarBase v3.0 database (see Footnote 6), and Cytoscape was used to visualize the mRNA-RBP regulatory network.

Finally, the Comparative Toxicogenomics Database^7^ was used to predict the indirect and direct drug targets of HRDEGs, investigate the interactions between HRDEGs and drugs, and visualize the mRNA-drug regulatory network using Cytoscape, completing the construction of the regulatory network.

### 2.9 Expression difference analysis of key genes

To identify the potential mechanisms of differential genes in SCI and their associated biological features and pathways, the Mann–Whitney U test was employed to analyze the expression differences of key genes between the SCI and control groups in the Combined Datasets. The differential analysis results were visualized using the R package ggplot2. A group comparison chart was generated.

ROC curves can be utilized to identify the best model, eliminate suboptimal ones, or determine the optimal threshold within the same model (Mandrekar, 2010). They represent sensitivity and specificity as continuous variables, demonstrating their relationship through composition. The AUC typically ranges from 0.5 to 1, with values closer to 1 signifying better diagnostic accuracy. AUC values between 0.5 and 0.7 indicate low accuracy, 0.7 and 0.9 indicate moderate accuracy, and above 0.9 indicate higher accuracy. Based on the results of differential expression analysis, ROC curves for these key genes were generated using the R package pROC. The AUC was calculated to assess the diagnostic effectiveness of HRDEGs in determining the survival of SCI patients.

### 2.10 Construction of high- and low-risk groups for hypoxia

To quantify the relative abundance of each gene in the dataset sample, single-sample Gene-Set Enrichment Analysis (ssGSEA) was used. The R package GSVA (Version 1.50.0^8^) was employed to calculate the hypoxia score (Hs) of all samples based on the expression matrix of key genes in the Combined Datasets using the ssGSEA algorithm. Based on the median expression value of the Hs, SCI samples were then categorized into high-risk and low-risk groups.

### 2.11 Immune infiltration analysis

ssGSEA was applied to quantify the relative abundance of each immune cell type (Xiao et al., 2020). First, infiltrating immune cell types were labeled, such as activated dendritic cells, natural killer cells, regulatory T cells, activated CD8 + T cells, and gamma-delta T cells. The enrichment score calculated by ssGSEA represented the relative abundance of each immune cell type in each sample, yielding an immune cell infiltration matrix for the Combined Datasets. Subsequently, the R package ggplot2 (version 3.4.4) was used to generate a group comparison chart to illustrate the expression differences of immune cells between the low-risk and high-risk groups. Immune cells with significant differences between the groups were selected for further analysis. The correlation between immune cells was assessed using the Spearman correlation coefficient and visualized in a heatmap generated by the R package pheatmap. The correlation between key genes and immune cells was also analyzed using the Spearman algorithm, and the R package ggplot2 (version 3.4.4) was utilized to generate a correlation bubble chart displaying the results.

### 2.12 Experimental validation using qPCR

Total RNA was extracted from spinal cord tissue samples of C57BL/6 J mice (Vital River Laboratory Animal Technology Co., Ltd., Beijing, China) using TRIpure Total RNA Extraction Reagent (EP013, ELK Biotechnology, Wuhan, China). All experimental procedures received approval from the Animal Ethics Committee of the First Hospital of Jilin University. Mice were categorized into sham-operated (*n* = 3) and SCI groups (*n* = 3). In the SCI group, mice were anesthetized and underwent laminectomy at T9-T11 to expose the T10 spinal cord. A 10 g impactor inflicted a contusion injury (2.5 mm diameter, 2 mm depth) at the T10 segment. The establishment of the model was confirmed by immediate hindlimb spasticity and loss of muscle tone. The incision was closed under aseptic conditions, and the mice were returned to their cages post-recovery. Sham-operated mice received identical surgical exposure without spinal cord impact. Seven days post-injury, spinal cord tissues from the injury epicenter of SCI mice or the corresponding region of sham-operated mice were harvested and snap-frozen in liquid nitrogen for further analysis. cDNA synthesis was conducted using EntiLink™ 1st Strand cDNA Synthesis SuperMix (EQ031, ELK Biotechnology, Wuhan, China) according to the manufacturer’s protocol. Real-time PCR was performed on a QuantStudio 6 Flex System (Life Technologies, CA, USA) using EnTurbo™ SYBR Green PCR SuperMix (EQ001, ELK Biotechnology). Thermal cycling parameters included an initial denaturation at 95°C for 3 min, followed by 40 cycles at 95°C for 10 s, 58°C for 30 s, and 72°C for 30 s. *ACTIN* served as the reference gene for normalization, and relative gene expression was quantified using the 2^−ΔΔCT^ method (Supplementary Table S3).

### 2.13 Statistical analysis

R software (version 4.2.2) was utilized for data analysis and processing. The independent Student’s *t*-test compared normally distributed continuous variables between two groups unless otherwise specified. The Mann–Whitney U test was applied for variables not normally distributed. For comparisons across three or more groups, the Kruskal-Wallis test was employed. The correlation coefficient between different molecules was determined using Spearman correlation analysis. Unless otherwise indicated, all *p*-values were two-sided, with values under 0.05 considered statistically significant.

## 3 Results

### 3.1 Data assembly and correction

**Figure 1:**
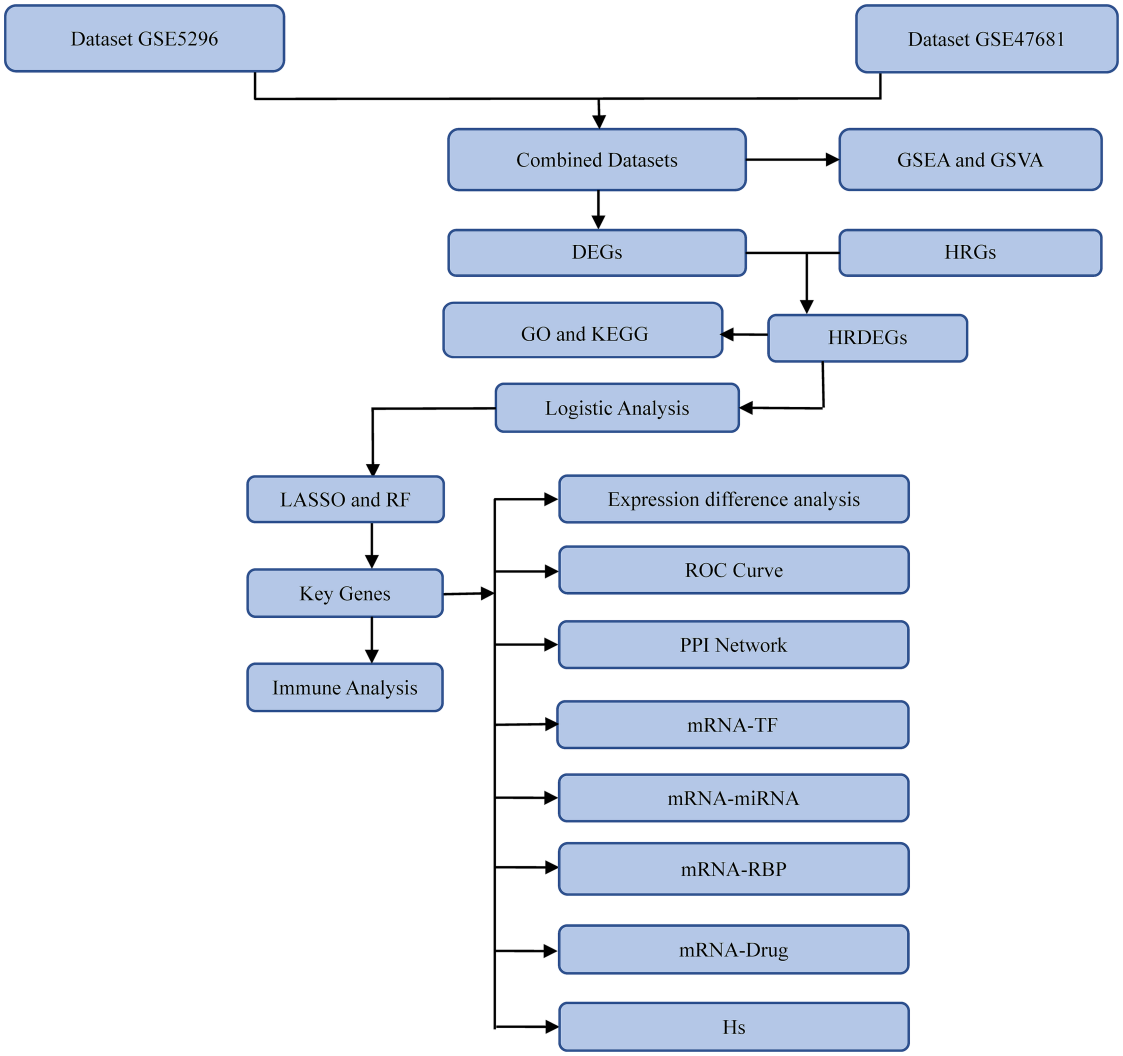
Technology roadmap. GSEA, Gene Set Enrichment Analysis; GSVA, Gene Set Variation Analysis; GO, Gene Ontology; KEGG, Kyoto Encyclopedia of Genes and Genomes; DEGs, Differentially expressed genes; HRGs, Hypoxia-related genes; HRDEGs, Hypoxia-related differentially expressed genes; LASSO, Least Absolute Shrinkage and Selection Operator; RF, Random Forest; ROC, receiver operating characteristic; PPI, protein–protein interaction; TF, Transcription factor; RBP, RNA-binding protein; Hs, Hypoxia Score.

**Figure 2:**
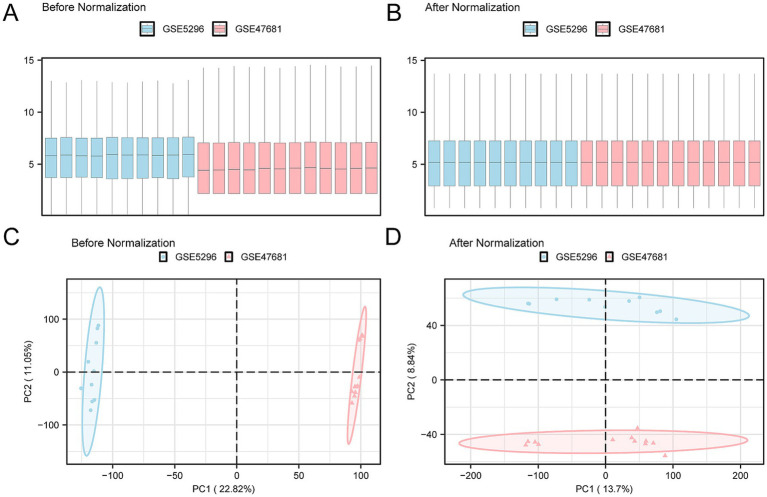
Batch effect removal of GSE5296 and GSE47681. **(A)** Box plot showing the distribution of expression values across the combined GEO datasets before batch processing. A significant difference is observed between the datasets GSE5296 (blue) and GSE47681 (pink), with GSE5296 generally exhibiting higher expression levels. This indicates that the two datasets are influenced by different degrees of technical variation due to distinct experimental conditions without batch effect removal. **(B)** Box plot showing the distribution of expression values in the integrated GEO dataset after batch processing. Following batch effect removal, the expression values of the two datasets become more consistent and nearly overlap, indicating successful batch effect removal. **(C)** PCA results of the pre-batch datasets. GSE5296 and GSE47681 are clearly separated in the low-dimensional feature space, suggesting significant differences and considerable variability between the datasets. **(D)** PCA plot of the integrated GEO dataset after batch effect removal. Post-batch processing, the samples in the principal component space show enhanced clustering and greater overlap, indicating increased similarity. GSE5296 (SCI dataset) is represented in blue, and GSE47681 (SCI dataset) is in pink. PCA, Principal Component Analysis; SCI, Spinal Cord Injury.

A technology roadmap illustrating the experimental design is depicted in Figure 1. Batch effects were eliminated from the SCI datasets GSE5296 and GSE47681, resulting in the Combined Datasets. The variations in expression values before and after the removal of batch effects are illustrated in distribution box plots (Figures 2A,B). Subsequently, the low-dimensional feature distribution of the datasets, both before and after batch effect removal, was compared using PCA diagrams (Figures 2C,D). These results indicate that the batch effects in the SCI datasets were effectively removed.

### 3.2 SCI-associated HRDEGs

**Figure 3:**
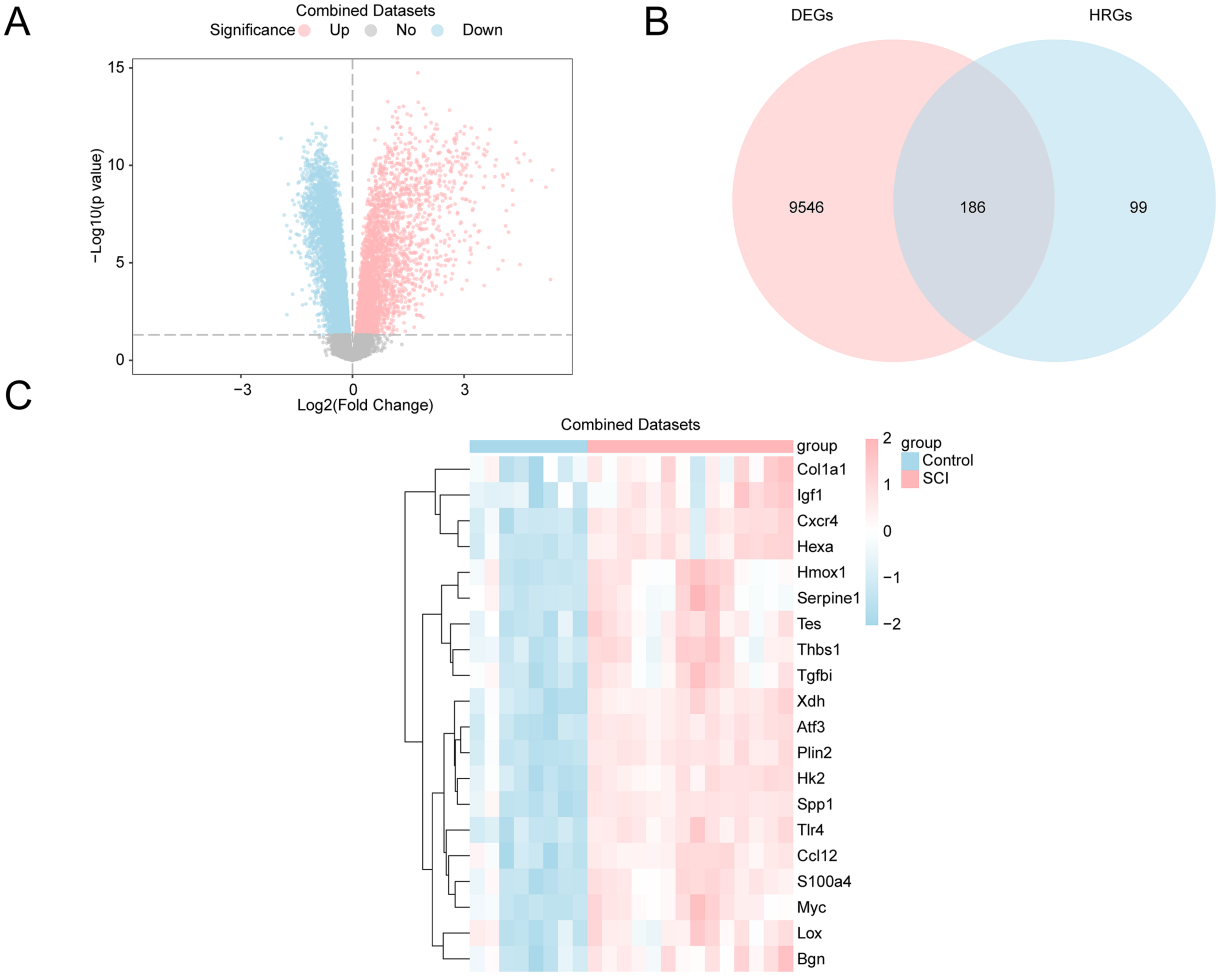
Differential gene expression analysis. **(A)** Volcano plot of differentially expressed genes (DEGs) in the SCI and control groups from the GEO dataset. Upregulated genes are shown in pink, down-regulated genes in blue, and non-significantly different genes in gray. The x-axis shows the log2 fold change, and the y-axis represents the negative logarithm of the *p*-value (−Log10(*p*-value)). **(B)** Venn diagram of DEGs and hypoxia-related genes (HRGs) in the GEO dataset, showing 186 genes that overlap as both DEGs and HRGs. **(C)** Heatmap of HRDEGs in the GEO dataset, illustrating gene expression levels in the SCI group versus the control group. The expression patterns of each gene are clearly shown, with pink indicating high expression in the SCI group and blue indicating low expression in the control group. SCI, Spinal Cord Injury; DEGs, Differentially Expressed Genes; HRGs, Hypoxia-Related Genes; HRDEGs, Hypoxia-Related Differentially Expressed Genes.

The Combined Datasets were categorized into two groups: SCI and control. The differential analysis identified 9,732 DEGs that met the threshold criteria of |logFC| > 0 and *p* < 0.05. Among them, 3,834 genes were upregulated, while 5,898 were downregulated. The differential analysis results are illustrated in a volcano plot (Figure 3A).

To identify HRDEGs, the intersection of all DEGs and HRGs with a |logFC| value of > 0 and a *p*-value of < 0.05 was taken, and a Venn diagram was created (Figure 3B). A total of 186 HRDEGs were obtained, including *Sap30, Plin2, Tlr4, Atf3, Jun, Kif5a, Pgm1, Xdh, Efna3, P4htm, Rhoa, Hk2, Slc2a3, Spp1, Atp7a, Bax, Pik3cg, Cxcr4, Hexa, Tpst2, Bnip3, Eno2, S100a4, Tnfaip3, CSCIp3, Cul2, Nedd4l, Ppargc1a, Myc, Mtor, CSCIp6, Nos1, Ccl12, Tpd52, Prkaa2, Pfkfb4, Klf6, Mapk8, Nfkb1, Pgm2, Tes, Rbpj, Myh9, Zfp36, Btg1, Slc6a6, Fos, Camk4, Anxa2, Hoxb9, Thbs1, Ddit3, Ldha, Gcnt2, Bgn, Siah2, Inha, Pygm, Ampd3, Cav1, Plaur, Galk1, Vegfa, Higd1a, Bhlhe40, Tgfbi, Lxn, Grhpr, Sdc4, Rest, Gaa, Kdelr3, Fosl2, Angptl4, Tgm2, Hmox1, Nagk, Egr1, Stbd1, Dcn, Col5a1, Kdm3a, Cp, Csrp2, Ugp2, Igf1, Pkm, Vldlr, Mmp2, Ets1, Pdgfb, Pdk3, Lox, Gapdh, Gpc4, F3, Ids, Pfkp, Stat3, Aldoa, Mdm2, Nfil3, Tpi1, Xpnpep1, Ext1, Car12, Serpine1, Nfe2l2, Tgfb1, Dpysl4, Hspa5, Rora, Ctnnb1, Hif1an, Scarb1, Sp1, Ackr3, Tiparp, Sdc3, Gpi1, Notch1, Pim1, Ak4, Egln1, Col1a1, Zfp292, Slc37a4, Hdlbp, Arnt2, Cxcl12, Plac8, Dtna, Rwdd3, Pdk1, Rragd, Bcan, Tmem45a, Maff, Ier3, Sult2b1, Hk1, Hipk2, Ptgs2, Ace, Trp53, Ndst1, Fgf2, Aldoc, Ncan, Kdr, Mif, Hsp90aa1, P4ha1, Dusp1, Ndst2, Cdkn1a, Selenbp1, Angpt2, Klf7, Chst2, Bcl2, Wsb1, Sdhb, Hilpda, Mapk1, Bdnf, Tnf, Gpc1, Gpc3, Sesn2, Higd2a, Nos3,Igfbp3, Pnrc1, Slc2a1, Egfr, Ddit4, Glrx, Eng, Nr3c1, Cited2, Th, Isg20, Sdc2, Ep300,* and *Arnt*. Based on the intersection results, the expression differences of HRDEGs between various sample groups were analyzed. The top 20 HRDEGs are shown in a heatmap (Figure 3C). The TOP20 genes were ranked based on their *p*-values, arranging from the smallest to the largest.

### 3.3 GO and KEGG pathway enrichment analyses

**Figure 4:**
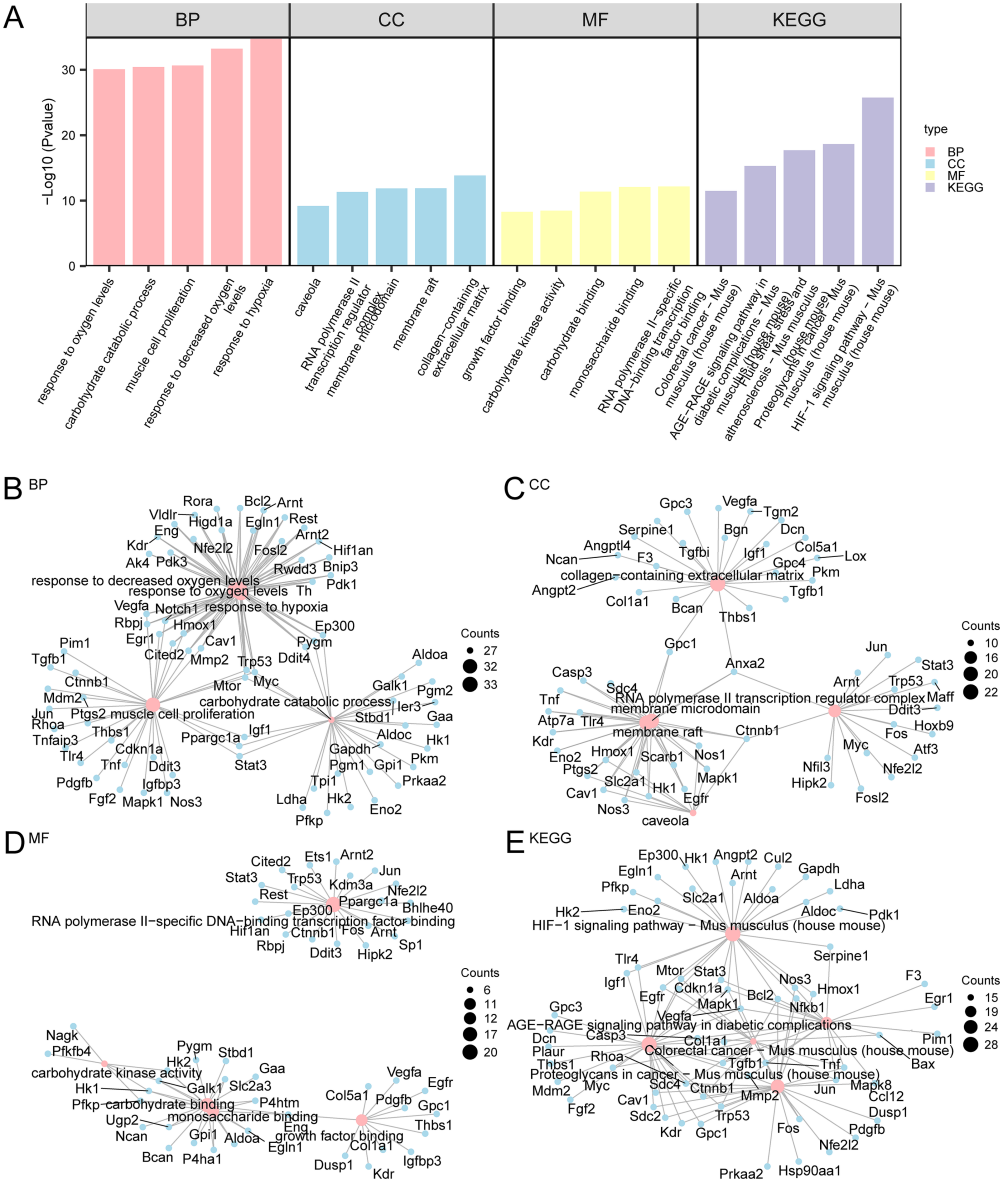
GO and KEGG pathway enrichment analyses for HRDEGs. **(A)** Bar graph showing the results of GO and KEGG pathway enrichment analysis of HRDEGs. The bars represent the significance of BP, CC, MF, and KEGG pathways. The height of each bar is the −Log10(*p*-value), where a higher value indicates greater statistical significance. Pink, blue, and yellow represent BP, CC, and MF, respectively, while purple represents KEGG pathways. **(B–E)** Network diagrams depicting the results of GO and KEGG pathway analyses for HRDEGs. Panel **(B)** represents BP, Panel **(C)** represents CC, Panel **(D)** represents MF, and Panel **(E)** shows KEGG pathways. In each diagram, pink nodes represent GO entries or KEGG pathways, blue nodes represent associated molecules, and lines indicate the relationships between entries and molecules. The GO and KEGG analyses were based on a *p*-value < 0.05 and FDR (*q*-value) < 0.05, with BH correction for *p*-values. HRDEGs, Hypoxia-Related Differentially Expressed Genes; GO, Gene Ontology; KEGG, Kyoto Encyclopedia of Genes and Genomes; BP, Biological Process; CC, Cellular Component; MF, Molecular Function.

GO and KEGG pathway enrichment analyses were conducted to further investigate the biological roles of the 186 HRDEGs. The MF, CC, and BP associated with these genes were explored. As shown in Supplementary Table S4, the 186 HRDEGs were primarily enriched in BPs, such as responses to decreased oxygen levels, hypoxia, muscle cell proliferation, carbohydrate catabolic process, and oxygen levels. Regarding CC, these genes were enriched in structures such as the collagen-containing extracellular matrix, RNA polymerase II transcription regulator complex, membrane microdomain, caveola, and membrane raft. The MF associated with these genes included RNA polymerase II-specific DNA-binding transcription factor binding, monosaccharide binding, carbohydrate binding, carbohydrate kinase activity, and growth factor binding. The KEGG pathway enrichment analysis revealed that these genes were also enriched in pathways such as the hypoxia-inducible factors (HIF-1) signaling pathway, fluid shear stress and atherosclerosis, proteoglycans in cancer, colorectal cancer, and the AGE-RAGE signaling pathway in diabetic complications. The GO and KEGG enrichment analysis results were visualized using histograms (Figure 4A). Additionally, network diagrams were created to visualize the BP, CC, MF, and KEGG pathways (Figures 4B–E).

### 3.4 Gene set enrichment (GSEA) and GSVA

**Figure 5:**
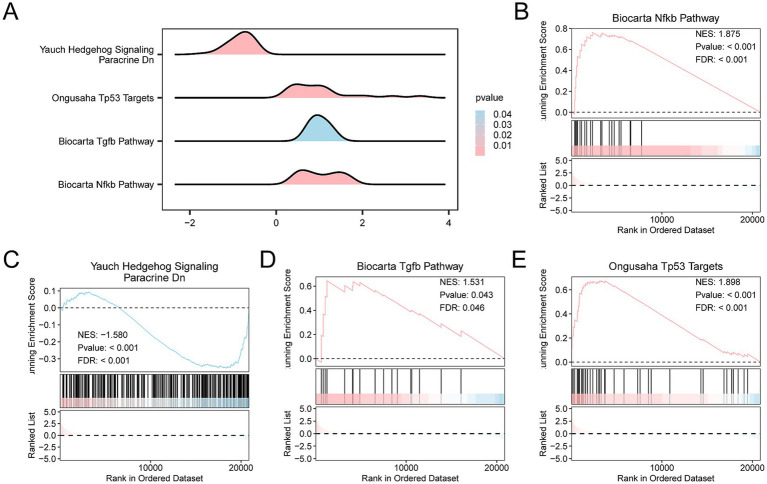
Differential gene expression analysis and GSEA for the Combined Datasets. **(A)** Gene Set Enrichment Analysis (GSEA) results for the GEO dataset, presented as mountain plots showing the enrichment of different gene sets. **(B–E)** GSEA results indicating significant enrichment of several biological pathways, including **(B)** Biocarta NF-kB Pathway, **(C)** Yauch Hedgehog Signaling, **(D)** Biocarta TGFβ Pathway, and **(E)** Ongusaha TP53 Targets. The color gradient in the mountain plots indicates statistical significance, with pink indicating smaller *p*-values and stronger statistical significance, and blue indicating larger *p*-values. **(B)** The Biocarta NF-kB Pathway had an normalized enrichment score (NES) of 1.875, *p*-value < 0.001, and FDR < 0.001, demonstrating strong enrichment. **(C)** The Yauch Hedgehog Signaling Pathway had an NES of −1.580, *p*-value < 0.001, and FDR < 0.001. **(D)** The Biocarta TGFβ Pathway had an NES of 1.531, *p*-value = 0.043, and FDR = 0.04. **(E)** The Ongusaha TP53 Targets Pathway had an NES of 1.898, *p*-value < 0.001, and FDR < 0.001. GSEA criteria were *p*-value < 0.05 and FDR (*q*-value) < 0.05, with BH correction applied.

GSEA was applied to the Combined Datasets to determine the impact of gene expression levels on the incidence of SCI. The analysis focused on the expression levels of genes in the SCI group compared with the controls, specifically examining logFC values between these groups. GSEA explored BP, CC, and MF affected by gene expression levels. A mountain diagram shows the GSEA of four biological functions (Figure 5A). Detailed results are shown in Supplementary Table S5. The analysis revealed significant enrichment of genes in several key pathways and functions, including the Biocarta Nfkb Pathway (Figure 5B), Yauch Hedgehog Signaling Paracrine Dn (Figure 5C), Biocarta Tgfb pathway (Figure 5D), and the Ongusaha Tp53 Targets (Figure 5E).

**Figure 6:**
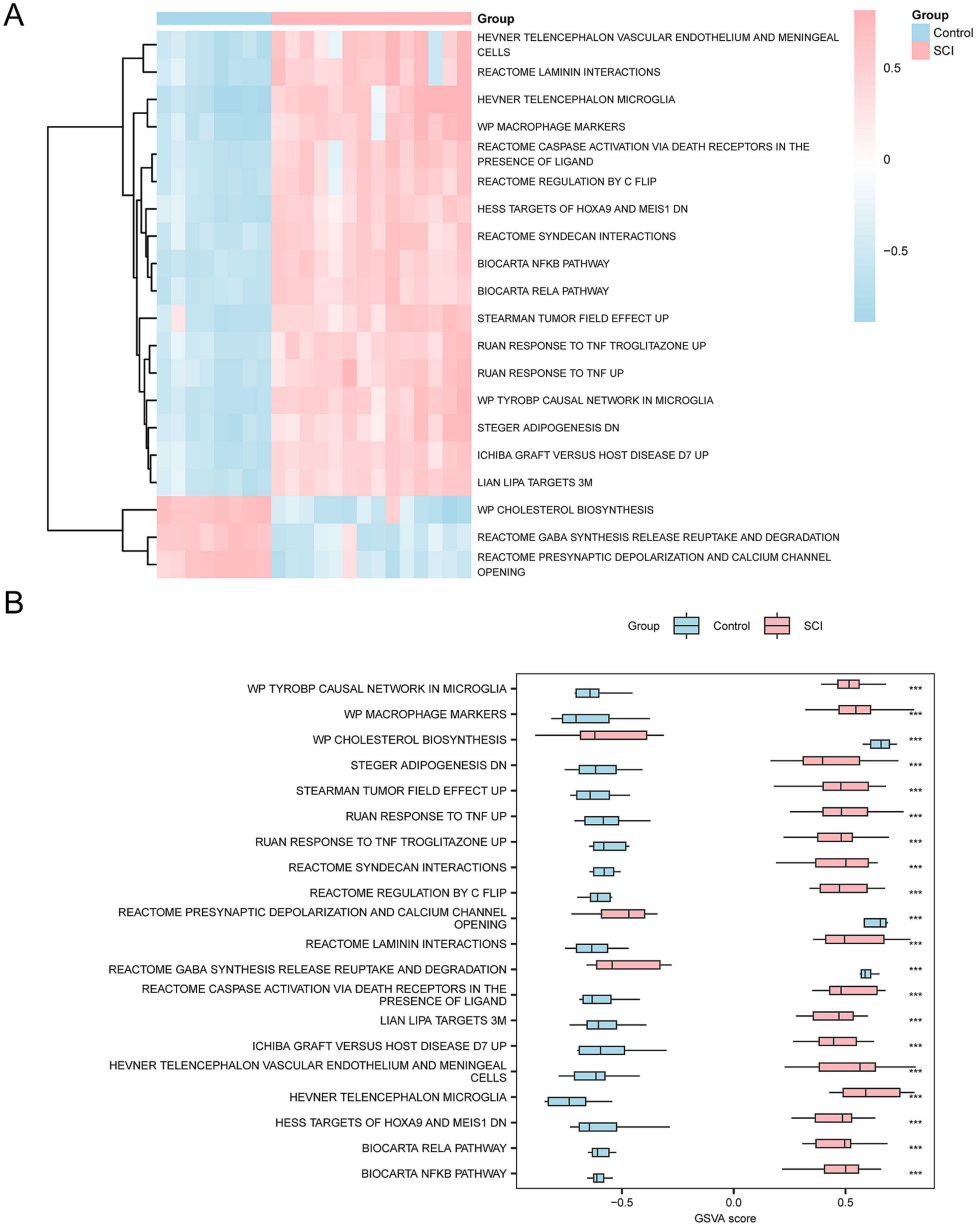
GSVA. **(A)** Heatmap showing the gene set variation (GSVA) between the SCI and control groups in the integrated GEO dataset. **(B)** Group comparison plot showing the variation of gene sets between the SCI and control groups. The heatmap visualizes the higher enrichment of gene sets in the SCI group (pink) compared to the control group (blue), and the group comparison plot indicates significant differences with *p*-value < 0.001. The GSVA criteria were *p*-value < 0.05, with BH correction applied. SCI, Spinal Cord Injury; GSVA, Gene Set Variation Analysis.

To explore the differences in the m2.all.v2023.2.Mm.symbols.gmt gene set between SCI patients and controls, GSVA was applied to all genes (Supplementary Table S6). Subsequently, the top 20 pathways with *p* < 0.05 were selected and ranked in descending order based on the absolute value of |logFC|. The differential expression of these 20 pathways between the SCI and control groups was analyzed and visualized in heatmaps (Figure 6A).

Subsequently, the differences between groups were verified using the Mann–Whitney U test. The results are shown in a group comparison chart (Figure 6B). The GSVA revealed several significant pathways in the SCI group compared to the controls (*p* < 0.05), including Tyrobp Causal Network in Microglia, Ruan Response to TNF Up, Ruan Response to TNF Troglitazone Up, Stearman Tumor Field Effect Up, Biocarta Rela Pathway, Biocarta Nfkb Pathway, Syndecan Interactions, Hess Targets of Hoxa9 and Meis1 Down, Regulation by c-Flip, Cscipscie Activation via Death Receptors in the Presence of Ligand, Macrophage Markers, Hevner Telencephalon Microglia, Reactome Laminin Interactions, and Hevner Telencephalon Vascular Endothelium and Meningeal Cells.

### 3.5 Construction of a diagnostic model for SCI

**Figure 7:**
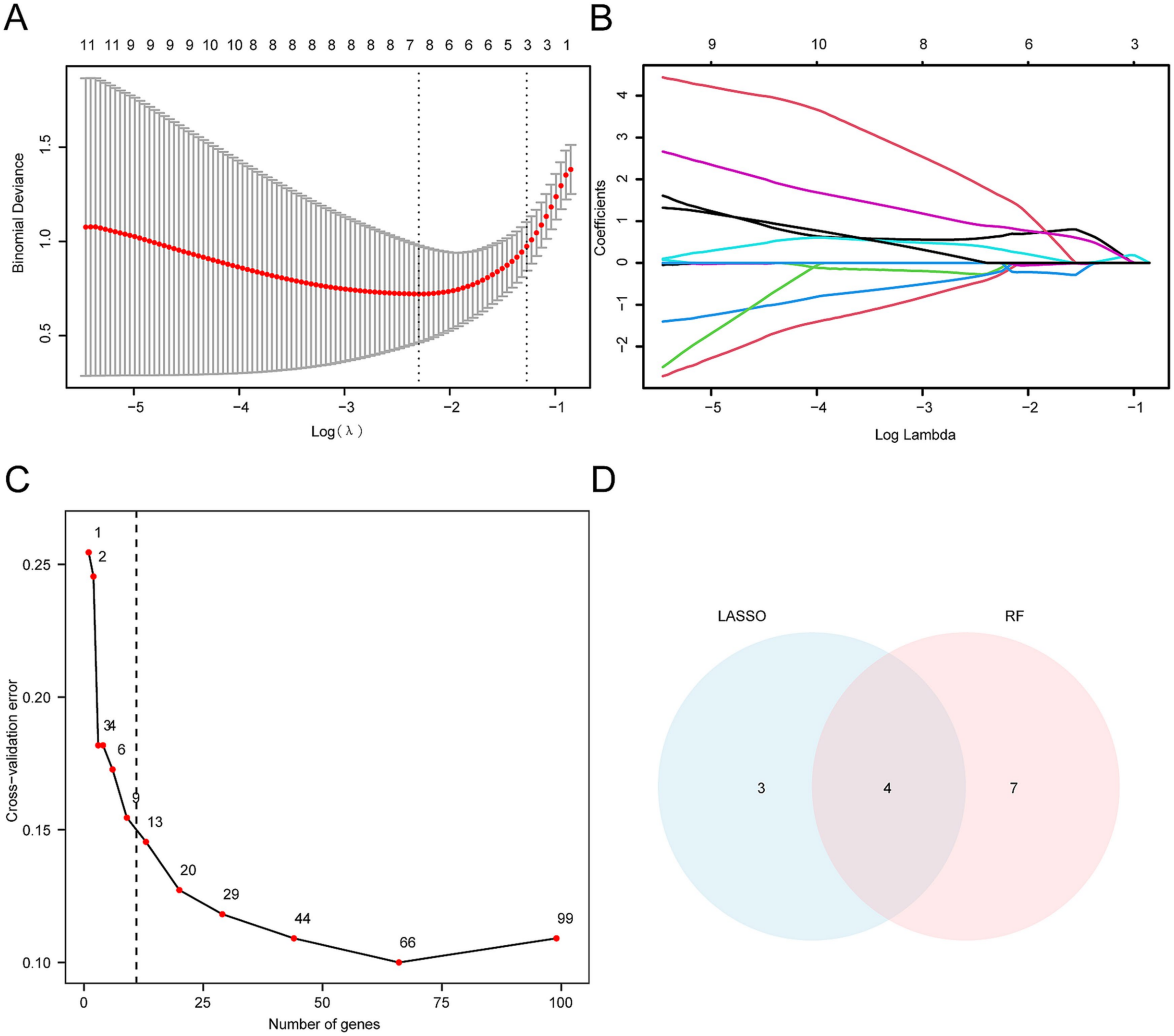
Construction of SCI diagnostic model. **(A)** LASSO regression diagnostic model diagram, constructed using HRDEGs from the integrated GEO dataset (Combined Datasets), illustrates the fit of the model. The red curve represents the optimal *λ* value (Log (λ)), which minimizes the corresponding binomial bias. **(B)** The variable trajectory diagram of the LASSO diagnostic model shows the changes in coefficients of different genes at various λ values. As λ increases, the coefficients of many genes progressively shrink and ultimately approach zero, indicating the model’s selectivity. **(C)** The cross-validation error curve demonstrates the change in model performance. The x-axis represents the number of genes, while the y-axis shows the cross-validation error. The red dot indicates the number of genes selected in the optimal model. The error decreases significantly as the number of genes increases and stabilizes once a certain threshold is reached. **(D)** The Venn diagram illustrating the intersection between the LASSO algorithm and the random forest algorithm (RF) shows the overlap of characteristic genes selected by both methods. The LASSO selects three unique genes, RF selects seven unique genes, and the intersection contains four genes.

A univariate logistic regression analysis was conducted to assess the diagnostic value of the 186 HRDEGs for SCI. Ninety-nine HRDEGs with statistical significance (*p* < 0.05) were identified (Supplementary Table S7). Subsequently, a LASSO regression analysis was performed using these 99 HRDEGs to develop a diagnostic model for SCI. The results were visualized through a LASSO regression model diagram (Figure 7A) and a LASSO variable trajectory plot (Figure 7B). The LASSO regression model highlighted seven HRDEGs as model genes, including *Casp6*, *Slc37a4*, *Pkm*, *Vldlr*, *Tiparp*, *Cxcr4*, and *Hexa*.

We used the RF algorithm to assess the diagnostic value of the 99 HRDEGs for SCI. The results showed that the error stabilized when the number of decision trees was 11 (Figure 7C). To refine the model, ten-fold cross-validation was performed five times, and a cross-validation error curve was plotted. The analysis revealed that the model error was minimized and stabilized when the number of genes was 11. The 11 HRDEGs that significantly impact the diagnosis of SCI are *Nagk*, *Hexa*, *Casp6*, *Pkm*, *P4ha1*, *Pdk3*, *Cav1*, *Angptl4*, *Cxcr4*, *Sdc3*, and *Galk1*.

To identify the key genes, we intersected the HRDEGs from the LASSO regression model with those identified by the RF algorithm. Four key genes (i.e., *Casp6*, *Pkm*, *Cxcr4*, and *Hexa*) were obtained and used for subsequent investigation. The intersection of these genes was visualized using a Venn diagram (Figure 7D).

### 3.6 Validation of the SCI diagnostic model

**Figure 8:**
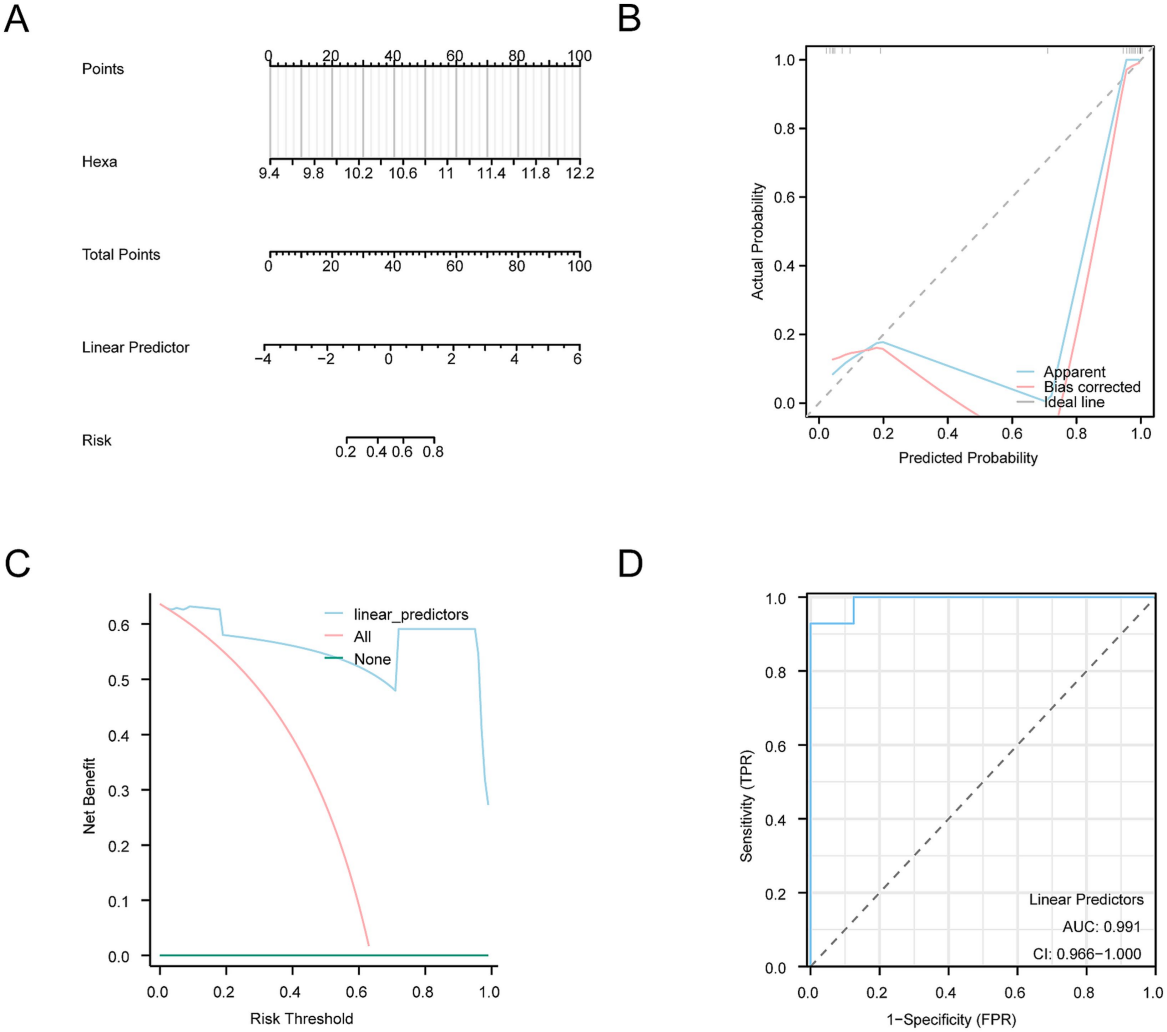
Diagnostic and validation analysis of SCI diagnostic model. **(A)** Nomogram of the integrated GEO dataset (Combined Datasets) shows how the probability of SCI is calculated based on identified risk factors. **(B)** The calibration curve shows the relationship between the predicted probability of SCI and the actual occurrence. The curve indicates how close the predicted probabilities are to the actual probabilities, whereas the bias correction curve represents the model’s adjusted prediction results, demonstrating the model’s reliability in prediction. **(C)** The DCA graph shows the net benefits at different risk thresholds. The y-axis represents the net benefit, and the x-axis represents the probability threshold. Different curves demonstrate the model’s performance at various thresholds, helping evaluate its potential benefits in decision-making. **(D)** ROC analysis of the linear predictor in the logistic regression model for the GEO dataset (Combined Datasets) displays the sensitivity and specificity of the model. The AUC was 0.991, indicating high accuracy, with an AUC value above 0.9. This figure shows the true positive rate (sensitivity) at different false positive rates (1-specificity). DCA, Decision Curve Analysis; ROC, Receiver Operating Characteristic; AUC, Area Under the Curve.

A nomogram was constructed based on the key genes identified to further substantiate the diagnostic value of the SCI model (Figure 8A). This nomogram elucidates the relationship between these genes and their contributions to the diagnostic model. After accounting for the missing genes, the expression level of the model gene *Hexa* showed significantly enhanced efficacy in diagnosing SCI.

A calibration analysis was conducted to assess the resolution and accuracy of the SCI diagnostic model. The calibration curve compares the actual probability of SCI with those predicted by the model under various conditions (Figure 8B). Although the dotted calibration line slightly deviated from the ideal diagonal, it remained closely aligned, indicating a well-calibrated model.

Furthermore, the clinical utility of the SCI diagnostic model was assessed using DCA based on the model genes from the Combined Datasets (Figure 8C). The model consistently provided higher benefits compared to the “All negative” and “All positive” strategies within a specified range, highlighting the robust predictive performance and clinical utility of the model.

ROC curves were subsequently generated to assess the diagnostic performance of the logistic regression model (Figure 8D). The results highlighted a strong diagnostic capability for the logistic regression model.

### 3.7 PPI network

**Figure 9:**
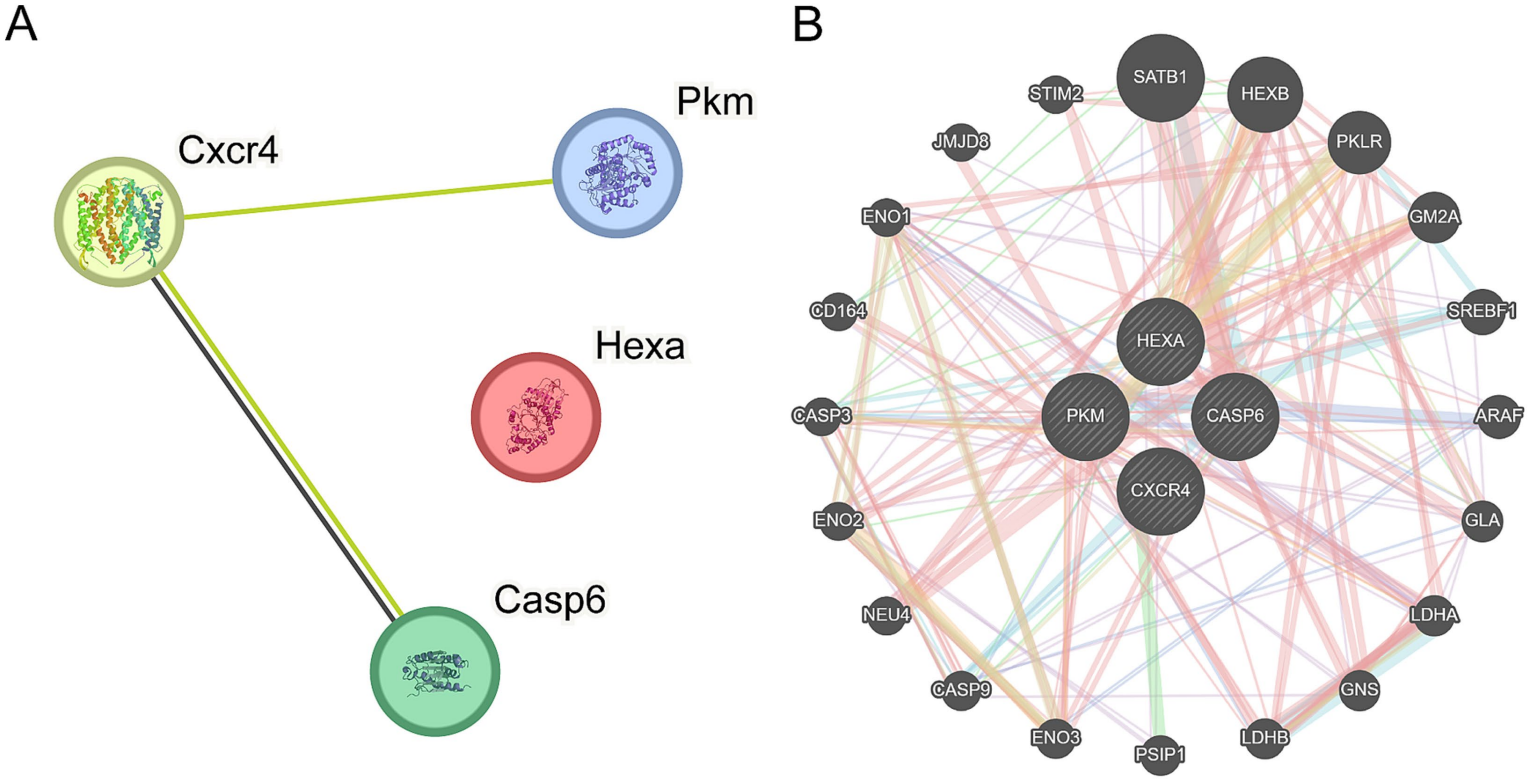
PPI network. **(A)** Protein–protein interaction (PPI) network of key genes shows interactions between genes such as *Cxcr4*, *Pkm*, *Hexa*, and *Casp6*. The size of the nodes correlates with the importance of each gene, whereas the connecting lines represent the interactions between genes. The number of lines reflects the strength of the interaction. **(B)** The interaction network of key genes predicting functionally similar genes reveals a broader range of gene interactions. Circular nodes represent genes, with their size reflecting their importance and influence within the network. The thickness of the connecting lines indicates the strength of the interactions.

PPI analysis for the four key genes (*Casp6*, *Pkm*, *Cxcr4*, and *Hexa*) was performed using the STRING database. A PPI network diagram is shown in Figure 9A. The GeneMANIA website was then used to predict genes similar to the key genes and create interaction networks. The shared protein domains and physical and gene interactions are shown in Figure 9B.

### 3.8 Construction of regulatory networks

**Figure 10:**
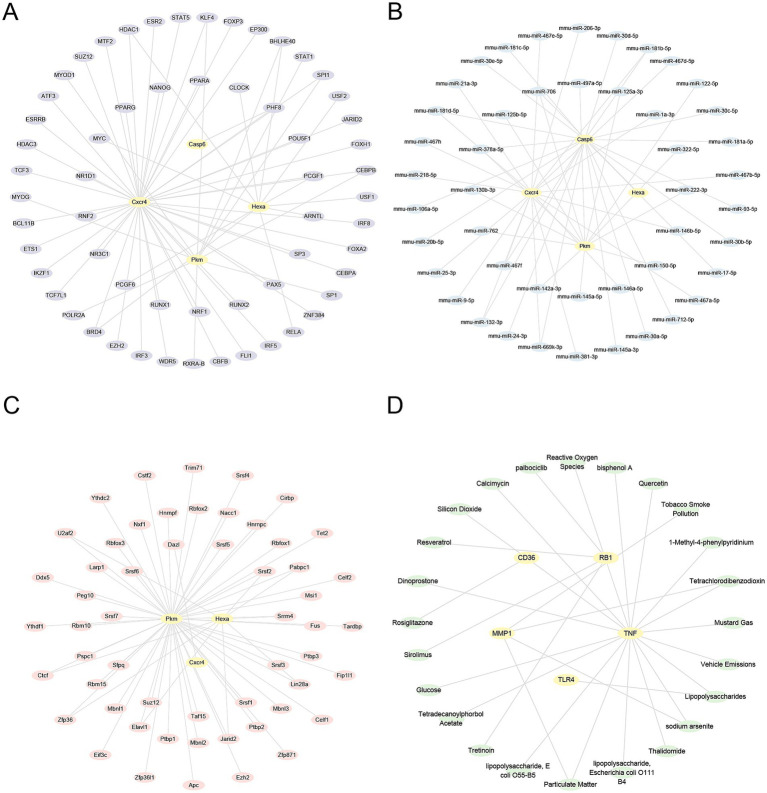
Regulatory networks of HRDEGs. **(A)** mRNA-TF regulatory network of HRDEGs illustrates the relationships between genes and transcription factors. Yellow nodes represent mRNA, and purple nodes represent transcription factors. **(B)** The mRNA-miRNA regulatory network of HRDEGs shows the interaction between mRNA and miRNAs. Yellow nodes represent mRNA, while blue nodes represent miRNAs. **(C)** The mRNA-RBP regulatory network of HRDEGs demonstrates the relationships between mRNA and RNA-binding proteins. Pink nodes represent RNA-binding proteins. **(D)** The mRNA-drug regulatory network of HRDEGs demonstrates the interactions with drugs. Green nodes represent drugs. HRDEGs, Hypoxia-Related Differentially Expressed Genes; TF, Transcription Factor; RBP, RNA-Binding Protein.

TFs associated with HRDEGs were identified using the ChIPBase database. The mRNA-TF regulatory network was then visualized using Cytoscape (Figure 10A). This network included four HRDEGs and 59 TFs (Supplementary Table S8). In addition, the miRNAs related to HRDEGs were identified through the TarBase and StarBase databases. The mRNA-miRNA regulatory network was constructed and visualized using Cytoscape (Figure 10B). This network included four HRDEGs and 46 miRNAs (Supplementary Table S9). Furthermore, RBPs related to HRDEGs were predicted using the StarBase database. These interactions were used to construct and visualize the mRNA-RBP regulatory network in Cytoscape (Figure 10C). There are three HRDEGs and 55 RBPs in this network (Supplementary Table S10). Furthermore, the Comparative Toxicogenomics Database was applied to identify potential molecular compounds or drugs related to HRDEGs. The mRNA-drug regulatory network was visualized in Cytoscape (Figure 10D). This network included one HRDEG and one drug or molecular compound (Supplementary Table S11).

### 3.9 Analysis of expression differences of key genes between different groups

**Figure 11:**
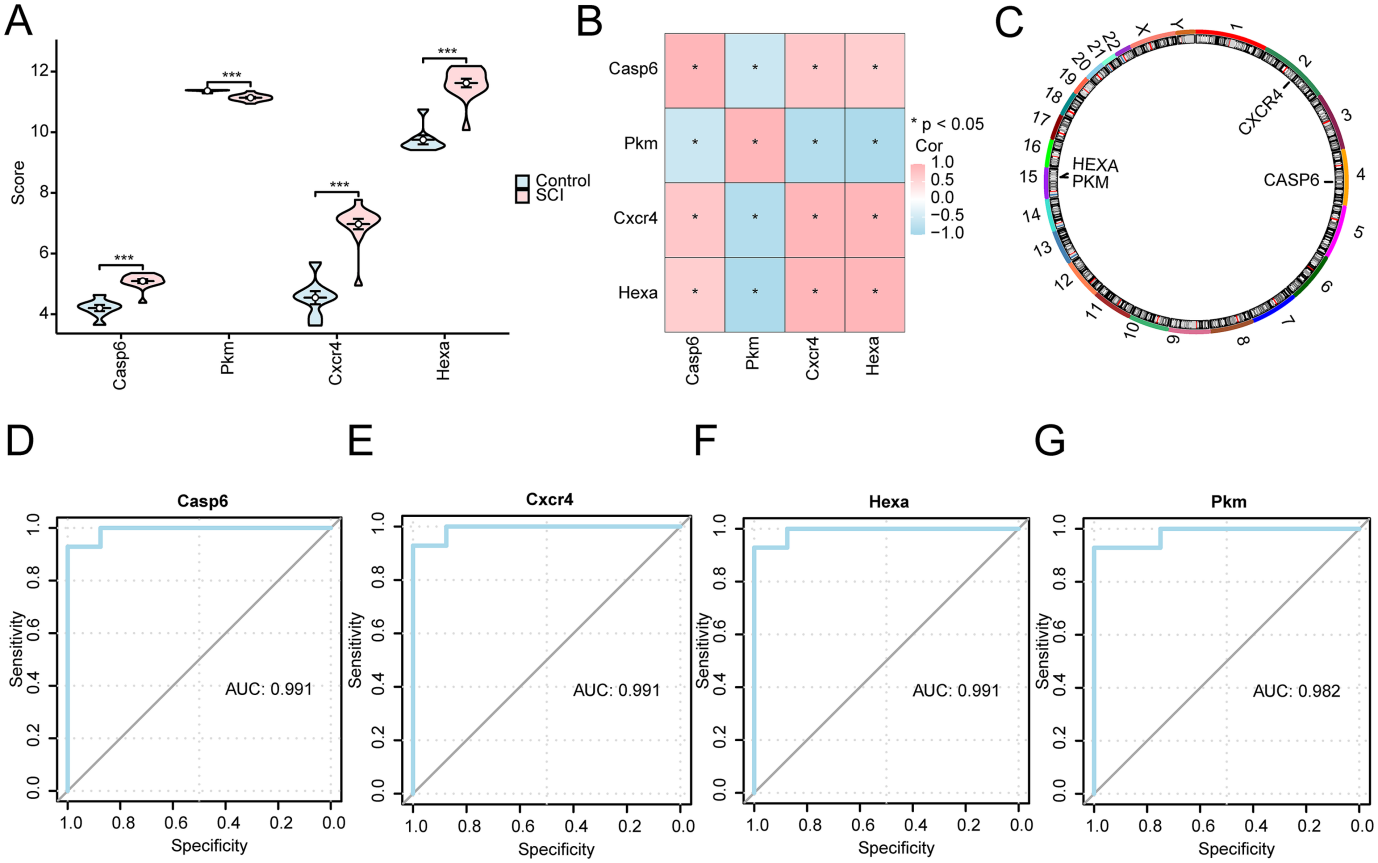
Analysis of expression differences of key genes between different groups. **(A)** Group comparison chart between the control and SCI groups shows the expression differences of key genes *Casp6*, *Pkm*, *Cxcr4*, and *Hexa*. Statistical symbols indicate *p* < 0.001, showing that the expression differences of these genes between the two groups are extremely statistically significant. In the figure, the pink box plot represents the SCI group, and the blue box plot represents the control group. **(B)** The correlation analysis between key genes is displayed by heat mapping, showing that these genes are significantly correlated. Correlation values above 0.5 in the heatmap are represented by color shades, where *p* < 0.05 indicates a significant positive correlation between key genes. **(C)** The chromosomal mapping of key genes in the human body shows the distribution of *Cxcr4*, *Hexa*, *Pkm*, and *Casp6* on different chromosomes. **(D–G)** ROC curve analysis of key genes **(D)** *Casp6*, **(E)** *Cxcr4*, **(F)** *Hexa*, and **(G)** *Pkm* was conducted to evaluate the diagnostic ability of these genes in spinal cord injury. The area under the curve (AUC) was 0.991 (*Casp6*, *Cxcr4*, and *Hexa*) and 0.982 (*Pkm*), indicating very high diagnostic accuracy for these genes. When the AUC value is close to 1, it indicates a better diagnostic effect; when the AUC value is above 0.9, it is considered to have higher accuracy.

The expression levels of four key genes (*Casp6*, *Pkm*, *Cxcr4*, *Hexa*) were significantly different in the SCI group versus controls (all *p* < 0.001; Figure 11A). Based on the complete expression matrix of the four key genes, correlation analysis was performed. *Casp6*, *Cxcr4*, and *Hexa* were all positively correlated, but *Pkm* was negatively correlated with other genes. (Figure 11B). Next, we converted the four key genes into their human orthologs and annotated their respective positions on human chromosomes to visualize them in a circle diagram (Figure 11C). The key gene *Cxcr4* is located on chromosome 2, *Casp6* on chromosome 4, and *Hexa* and *Pkm* on chromosome 15. The ROC curves of these genes are shown in Figures 11D–G. Their expression differences exhibit high accuracy, with all genes achieving an AUC greater than 0.9, indicating their strong potential as diagnostic markers.

### 3.10 Construction of high- and low-risk groups for hypoxia

**Figure 12:**
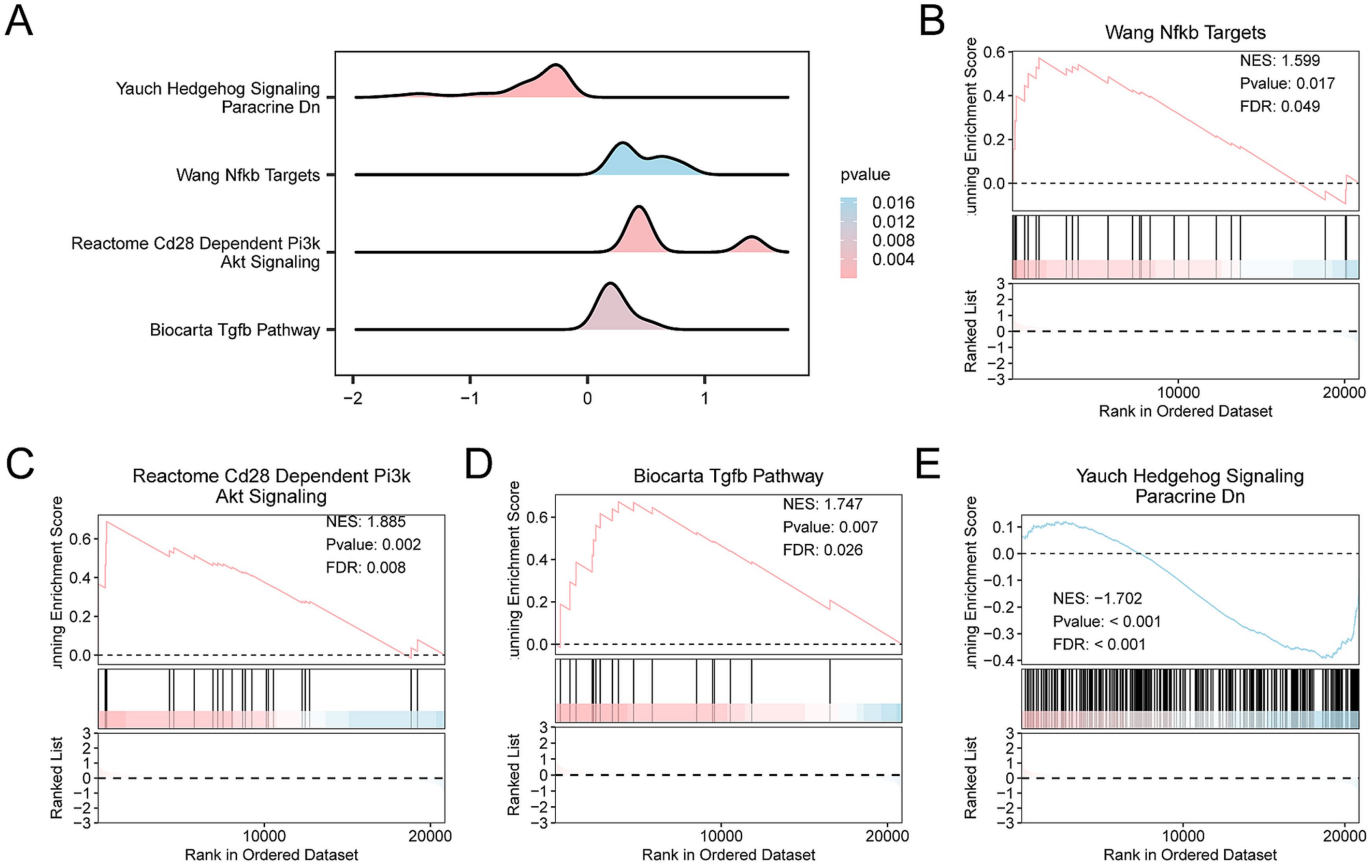
Differential gene expression analysis and GSEA for the Combined Datasets. **(A)** GSEA of the integrated GEO dataset (Combined Datasets) demonstrates four important biological functions through mountain maps. In the mountain diagram, the depth of color represents the magnitude of the *p*-value. The pinker the color, the smaller the *p*-value, indicating greater statistical significance, while the bluer the color, the larger the *p*-value. **(B–E)** The results of GSEA revealed that the integrated GEO dataset was significantly enriched in the following biological pathways. **(B)** Wang Nfkb Targets with a NES of 1.599, *p*-value of 0.017, and FDR of 0.049. **(C)** Reactome Cd28 Dependent Pi3k Akt Signaling with an NES of 1.885, *p*-value of 0.002, and FDR of 0.008. **(D)** Biocarta Tgfb pathway with an NES of 1.747, *p*-values of 0.007, and FDR of 0.026. **(E)** Yauch Hedgehog Signaling Paracrine Dn with an NES of −1.702, *p*-value < 0.001, and FDR < 0.001.

The Hs of all samples was calculated using the ssGSEA algorithm based on the levels of the four key genes. The samples were then categorized into high-risk and low-risk groups based on the median Hs of SCI samples. A mountain diagram was plotted to display GSEA results (Figure 12A). The genes in the Combined Datasets were significantly enriched in the Wang Nfkb Targets pathway (Figure 12B), the Reactome Cd28 Dependent Pi3k Akt Signaling pathway (Figure 12C), the Biocarta Tgfb pathway (Figure 12D), and the Yauch Hedgehog Signaling Paracrine Dn pathway (Figure 12E).

**Figure 13:**
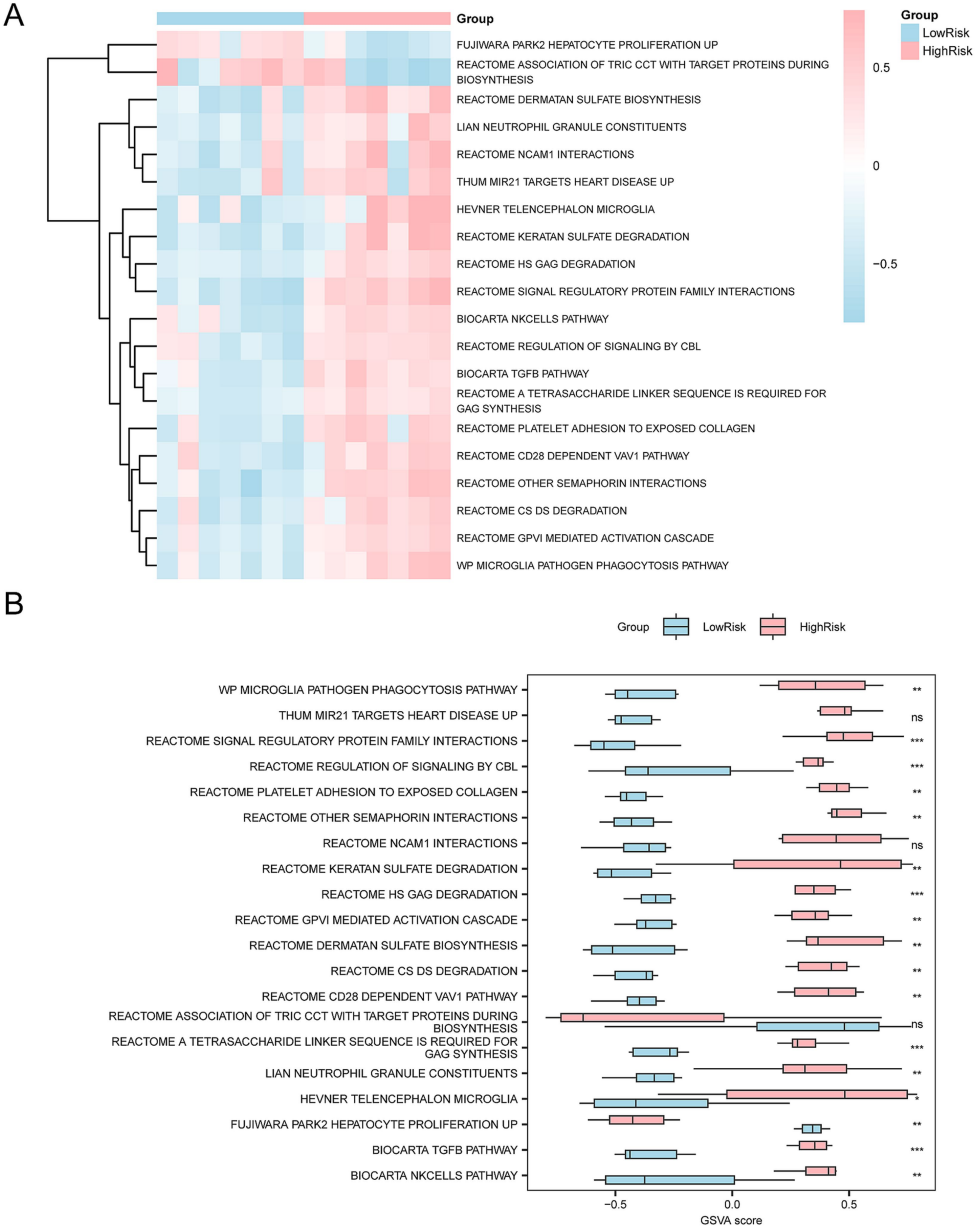
GSVA. **(A,B)** In the integrated GEO datasets (Combined Datasets), GSVA showed significant differences between the high-risk and low-risk groups. **(A)** The heatmap visualizes the enrichment of different gene sets between the two groups. The pink regions in the high-risk groups represent higher GSVA scores in multiple specific biological pathways, whereas the blue regions in the low-risk groups indicate lower enrichment in these pathways. **(B)** The group comparison plot further quantifies the difference in gene set enrichment between the two groups, and the GSVA scores of each pathway are contrasted by bar graphs. *ns* represents a *p*-value ≥ 0.05, showing no statistical significance; represents a *p*-value < 0.05 indicating a significant difference; indicates a *p*-value < 0.01, showing high statistical significance; then indicates a *p*-value < 0.001, indicating great statistical significance. The screening criteria for GSVA were *p*-value < 0.05, and the *p*-value correction method used was Benjamini-Hochberg (BH).

To explore the differences between the high-risk and low-risk groups, we conducted GSVA on all genes from the Combined Datasets. The top 20 pathways with *p* < 0.05 were sorted in descending order of logFC absolute. The differential expression of these pathways was visualized using heatmaps (Figure 13A). The difference was validated using the Mann–Whitney U test and a comparison chart (Figure 13B). The following pathways were significantly different between the two groups (*p* < 0.05): Microglia pathogen phagocytosis pathway, Reactome GPVI mediated activation cascade, Reactome CS DS degradation, Reactome other semaphorin interactions, Reactome CD28 dependent VAV1 pathway, Reactome platelet adhesion to exposed collagen, Reactome A tetrasaccharide linker sequence is required for GAG synthesis, Biocarta Tgfb pathway, Reactome regulation of signaling by CBL, Biocarta NK cells pathway, Reactome signal regulatory protein family interactions, Reactome HS GAG degradation, Reactome keratan sulfate degradation, Hevner telencephalon microglia, Li AN neutrophil granule constituents, Reactome dermatan sulfate biosynthesis, Fujiwara PARK2 hepatocyte proliferation up.

### 3.11 Immune infiltration analysis

**Figure 14:**
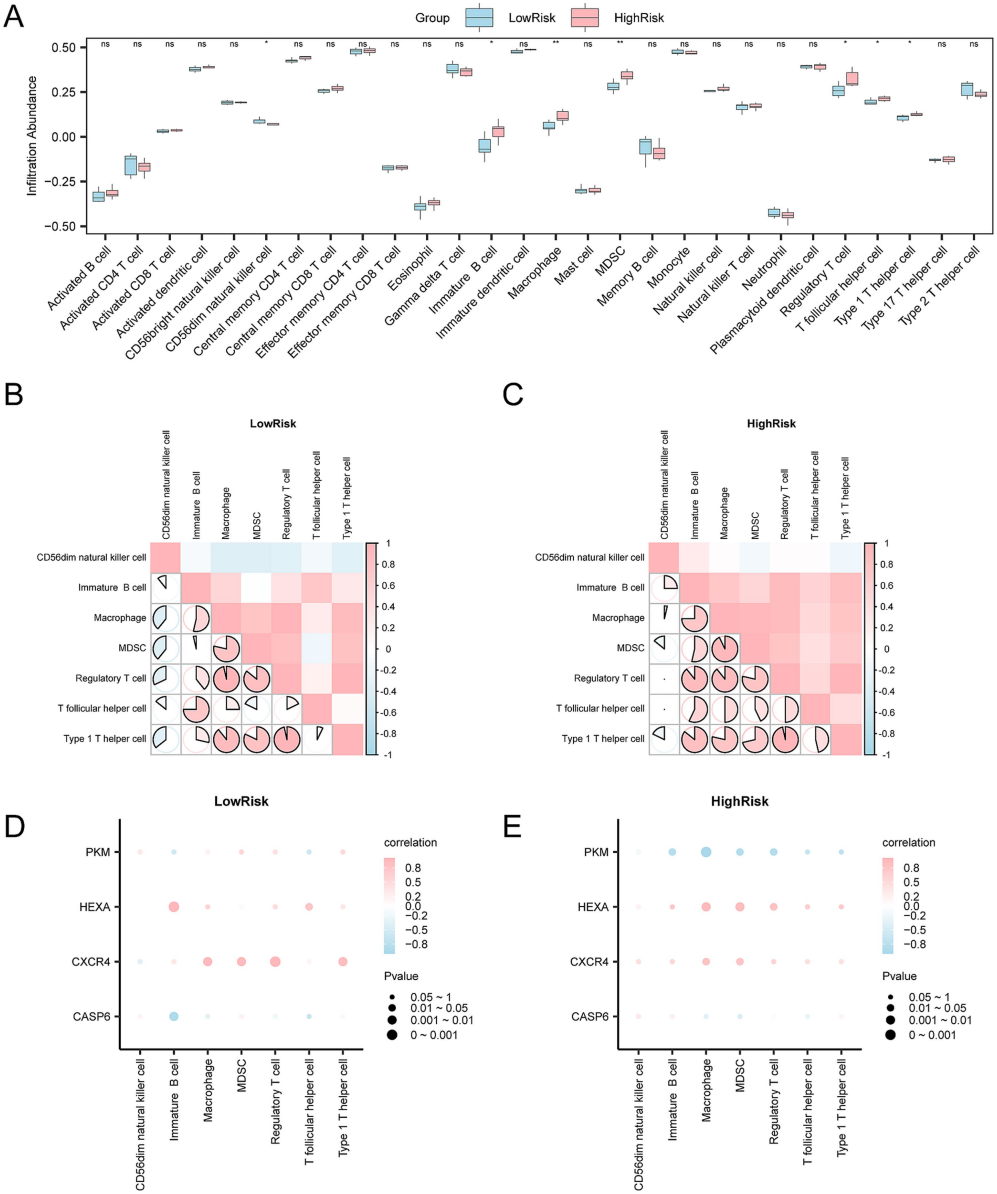
Risk group immune infiltration analysis by ssGSEA algorithm. **(A)** The figure shows the abundance changes of each immune cell type, with blue representing the low-risk group and pink representing the high-risk group. *ns* represents no statistical significance (*p*-value ≥ 0.05), indicates statistical significance (*p*-value < 0.05), and indicates higher significance levels (*p*-values < 0.01 and < 0.001, respectively). **(B,C)** The correlation analysis results of the **(B)** low-risk and **(C)** high-risk groups show the correlation of infiltration abundance between different immune cells. The bright red area in the heatmap indicates a positive correlation, while the blue area indicates a negative correlation. The depth of color reflects the strength of the correlation. The range of absolute values of correlation coefficients (*r*-values) illustrates the correlation of immune cells within each group. **(D,E)** In the **(D)** low-risk and **(E)** high-risk groups, the bubble chart further demonstrates the correlation between immune cell infiltration abundance and key genes. The bubble size is proportional to the intensity of the correlation, and the color change indicates the direction of the correlation. Pink indicates a positive correlation, and blue indicates a negative correlation. The absolute value of the correlation coefficient (*r*-value) is weak or uncorrelated below 0.3, weakly correlated between 0.3 and 0.5, moderately correlated between 0.5 and 0.8, and strongly correlated above 0.8. Blue indicates the low-risk group, and orange indicates the high-risk group.

The expression matrix of the Combined Datasets was converted into a human matrix. The immune infiltration abundance of 28 types of immune cells in SCI samples was then calculated using the ssGSEA algorithm. As shown in the group comparison diagram, seven types of immune cells were significantly different between groups (*p* < 0.05), including Type 1 T helper cells, T follicular helper cells, myeloid-derived suppressor cells, immature B cells, CD56dim natural killer cells, macrophages, and regulatory T cells (Figure 14A). The correlation results of immune cells infiltration abundance in SCI samples are shown in a correlation heatmap (Figures 14B,C). In the low-risk group, most immune cells exhibited strong correlations, with Type 1 T helper cells and regulatory T cells showing the strongest positive correlation (*r* = 0.964, *p* < 0.05) (Figure 14B). In the high-risk group, most immune cells exhibited strong positive correlation, with regulatory T cells and Type 1 T helper cells showing the strongest positive correlation (*r* = 0.964, *p* < 0.05) (Figure 14C). Finally, the correlation bubble chart shows the relationship between key genes and immune cell infiltration abundance (Figures 14D,E). In the low-risk group, most immune cells showed a strong positive correlation, with the gene *Cxcr4* and regulatory T cells demonstrating the strongest positive correlation (*r* = 0.964, *p* < 0.05) (Figure 14D). In the high-risk group, the gene *Pkm* and macrophages had the strongest negative correlation (*r* = −0.964, *p* < 0.05) (Figure 14E).

### 3.12 Experimental validation of key genes

**Figure 15:**
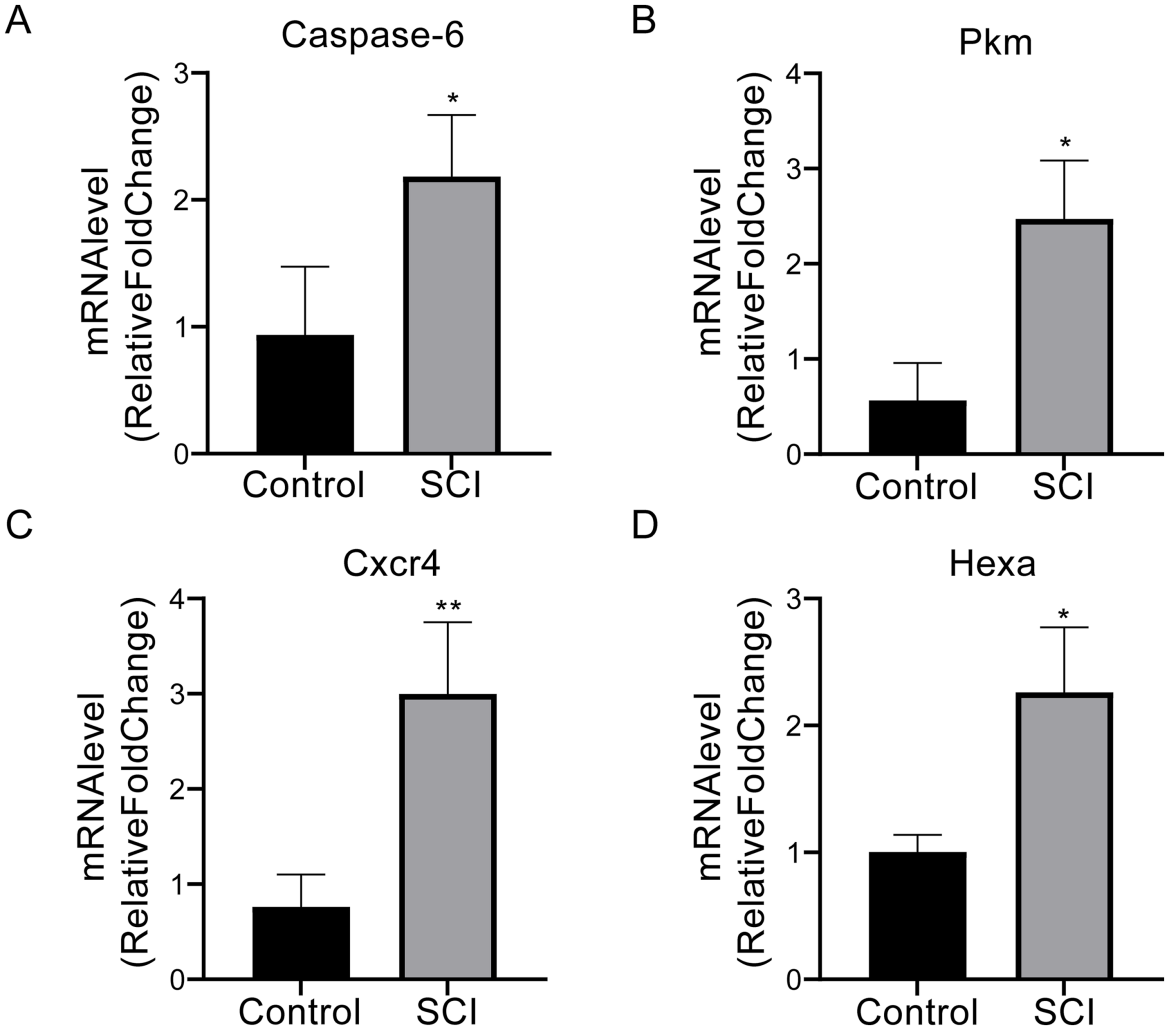
The qPCR results for *Casp6*, *Pkm*, *Cxcr4*, and *Hexa* in SCI and control groups. **(A)** Casp6 (apoptosis-related protease) expression was significantly elevated in the SCI group (2.18 ± 0.49) compared to sham controls (0.94 ± 0.54; *p* < 0.05). **(B)** SCI mice exhibited increased expression of the metabolic regulator Pkm (2.47 ± 0.61), surpassing sham-operated levels (0.57 ± 0.39; *p* < 0.05). **(C)** Cxcr4 (inflammatory chemokine receptor) displayed the most pronounced intergroup difference (3.00 ± 0.75 vs. 0.76 ± 0.34; *p* < 0.01). **(D)** Lysosomal enzyme Hexa was markedly upregulated post-injury (2.26 ± 0.52 vs. 1.00 ± 0.13; *p* < 0.05). Data are presented as mean ± SD. *p* < 0.05 vs. sham-operated group. *p < 0.05, **p < 0.01. SCI, spinal cord injury.

The mean expression level of *Casp6* was significantly lower in the sham-operated group compared to the SCI group (0.94 ± 0.54 vs. 2.18 ± 0.49, *p* < 0.05) (Figure 15A). Similarly, *Pkm* expression was markedly lower in the sham-operated group than in SCI mice (0.57 ± 0.39 vs. 2.47 ± 0.61, *p* < 0.05) (Figure 15B). *Cxcr4* exhibited notably lower levels in sham-operated mice compared to the SCI group (0.76 ± 0.34 vs. 3.00 ± 0.75, *p* < 0.01) (Figure 15C). Additionally, *Hexa* expression was significantly higher in SCI mice than in their sham-operated counterparts (2.26 ± 0.52 vs. 1.00 ± 0.13, *p* < 0.05) (Figure 15D). These results indicate that SCI significantly alters the expression of these genes compared to the sham-operated group.

## 4 Discussion

SCI is a significant public health concern affecting approximately 250,000 to 500,000 individuals globally annually. It severely impairs physical functions, significantly impacts patients’ quality of life, and imposes a substantial socioeconomic burden (Liang et al., 2022; Hu et al., 2023). Current treatments for SCI, including surgical intervention, medication, and rehabilitation, aim to mitigate secondary damage and promote recovery. While surgery can alleviate spinal compression, the regenerative capacity of damaged neurons remains limited, and the procedure itself carries risks such as infection or further injury. Medications primarily reduce inflammation and edema, but their effects on nerve regeneration and functional recovery remain limited. Furthermore, individual differences in drug efficacy and long-term use may lead to side effects. Although rehabilitation therapy can enhance patients’ daily living abilities, its impact on neural function recovery is limited, requiring a long-term and continuous treatment process. Therefore, in-depth studies on the pathogenesis of SCI and identifying novel therapeutic targets and biomarkers are crucial for improving the prognosis of patients with SCI. Recent studies have identified various genes and pathways involved in SCI, including those related to autophagy (Shang et al., 2024) and ferroptosis (Qu et al., 2023). However, a comprehensive analysis of HRDEGs and their regulatory networks in SCI remains largely unexplored (Ahuja et al., 2017). Furthermore, there is a notable lack of comprehensive analyses integrating multiple datasets to construct diagnostic models and explore protein interaction networks, particularly those focusing on the relationship between regulatory networks and immune infiltration in SCI.

SCI is a complex pathological condition influenced by numerous factors, including the type and location of the injury and subsequent inflammatory and immune responses (Hu et al., 2023). Although our sample size is limited, all samples in this study were obtained from *Mus musculus*. Additionally, to maintain consistency in injury models (contusion injury), we selected datasets GSE5296 and GSE47681. To further ensure uniformity in injury and tissue damage assessments, we conducted batch effect removal using the R package sva (version 3.50.0). This method facilitates cross-dataset comparisons by eliminating technical variations.

In this study, we identified 3,834 upregulated genes and 5,898 downregulated genes in the SCI group compared to the control group. Among them, 186 HRDEGs were identified. Activating transcription factor 3 (encoded by *Atf3*) is significantly upregulated under hypoxic conditions, where it protects nerve cells from damage by regulating apoptosis and autophagy, consistent with the findings by Liang et al. (2020). Toll-like receptor 4 (encoded by *Tlr4*) is implicated in inflammatory responses, and its upregulation may promote immune cell activation, facilitating debris clearance and tissue repair at injured sites, indirectly supporting nerve cell regeneration (Muendlein et al., 2022). These results validate the reliability of our study. Heme oxygenase-1 (encoded by *Hmox1*), an enzyme that catalyzes heme degradation, exerts cytoprotective effects and enhances neuronal survival under hypoxic stress (Liu et al., 2022). SERPINE1 regulates proteolysis and extracellular matrix remodeling, which is crucial in tissue repair (Akbar et al., 2023). Collagen Type I alpha 1 chain (encoded by *Col1a1*) contributes to extracellular matrix formation and tissue scaffolding, offering structural support essential for neuronal regeneration (Dong et al., 2021). Insulin-like growth factor 1 (encoded by *Igf1*) promotes cell growth and differentiation, acting as a key factor in neuronal repair and regeneration (Bailes and Soloviev, 2021). C-X-C chemokine receptor Type 4 (encoded by *Cxcr4*) facilitates cell migration and homing, potentially recruiting reparative cells to the injury site (Khasawneh and Abu-El-Rub, 2022). Additionally, the upregulation of *Tes*, *Thbs1*, and *Tgfbi* suggests their involvement in cell signaling, angiogenesis, extracellular matrix remodeling, and metabolic processes, contributing to the regenerative responses (Wang et al., 2019; Isenberg and Roberts, 2020; Huang et al., 2024). Together, these genes form a coordinated network that promotes neuronal survival and regeneration, which aligns with our results.

This study identified a series of genes closely related to SCI. GO and KEGG enrichment analyses provided valuable insights into the underlying signaling pathways and BPs. Notably, the significant enrichment of the HIF-1 signaling pathway offers a new perspective for understanding the pathological process of SCI. HIF-1, a critical transcription factor, plays a central role in cellular adaptive responses to hypoxic environments. HIF-1 is a central hub of hypoxia signaling, coordinating cellular metabolism, angiogenesis, and survival adaptation through the transcriptional regulation of HRDEGs. It plays a key role in tumors, ischemic diseases, and chronic inflammation. Following SCI, the affected spinal cord region often experiences local hypoxia, leading to activation of the HIF-1 signaling pathway (Huang et al., 2022). HIF-1 promotes angiogenesis, erythropoiesis, and reprogramming of energy metabolism by regulating the expression of downstream target genes, such as vascular endothelial growth factor and erythropoietin, thereby facilitating blood supply restoration and oxygenation capacity enhancement in damaged tissues (Huang et al., 2022). These adaptive responses are critical for tissue repair and nerve regeneration after SCI.

Therefore, further studies of the specific mechanisms of the HIF-1 signaling pathway in SCI are warranted. In addition to HIF-1, we found significant enrichment of cancer-related pathways, particularly proteoglycans in cancer, which is intriguing. While SCI and cancer are distinct diseases, they may share common molecular mechanisms. For instance, the HIF-1 signaling pathway, critical for hypoxic adaptation in cancer, is also activated in the hypoxic environment of SCI, as reported by Song et al. (2023). Similarly, proteoglycans, key components of the extracellular matrix in cancer, play essential roles in regulating cell–cell interactions, signal transduction, and cell migration and are equally significant in tissue repair and remodeling following SCI (Yao et al., 2022). Oxidative stress, a process intimately linked to cancer development, is considered a critical mechanism driving pathological progression and apoptosis following SCI. Cells subjected to SCI undergo significant oxidative stress, resulting in cellular damage and the activation of pro-apoptotic signaling pathways. This response mirrors cellular stress mechanisms observed in cancer, where oxidative stress is a major contributor to tumor progression. Upon the occurrence of SCI, damaged nerve cells and surrounding tissues produce large quantities of reactive oxygen species, leading to an elevated oxidative state that impairs cell membranes, nucleic acids, and proteins. Oxidative stress causes direct cellular damage and triggers multiple signaling pathways, including the p53 and NF-κB pathways (Anjum et al., 2020). The p53 signaling pathway is pivotal in regulating apoptosis and maintaining genomic stability, whereas the NF-κB pathway is critical in mediating inflammatory responses (Anjum et al., 2020). Activation of these pathways influences apoptosis, inflammation, and regeneration, ultimately altering the microenvironment of the injured area, thereby exacerbating damage and impeding recovery. These insights may offer new directions for interdisciplinary research.

The results from GSEA and GSVA revealed that gene expression in SCI samples was significantly enriched in several key biological pathways, such as the NF-κB and TGF-*β* signaling pathways. These pathways are closely implicated in inflammation, immune responses, and tissue repair, highlighting their potential regulatory roles in SCI (Jing et al., 2021; Nakazaki et al., 2021). This study employed LASSO regression and the RF algorithm to identify key genes for the SCI diagnostic model. LASSO regression effectively reduces model complexity by selecting the most significant variables, simplifying subsequent biological validation and clinical application. Conversely, the RF algorithm provides a more comprehensive perspective for model construction by evaluating the combined effects of multiple variables. The complementarity of the two methods ensures the scientific rigor and practical applicability of the key gene screening process.

Among the key HRDEGs identified in this study, *Casp6*, *Pkm*, *Cxcr4*, and *Hexa* play vital roles in SCI pathogenesis, providing novel insights into potential therapeutic targets. Casp6, an effector caspase in the apoptosis pathway, is pivotal in neuronal cell death following SCI (Ramírez-Moreno et al., 2023). Its upregulation in SCI samples suggests a direct contribution to the neurodegeneration observed in SCI patients, consistent with a previous report linking Casp6 to neuronal apoptosis in neurological disorders (Ramírez-Moreno et al., 2023). These findings indicate that Casp6 may be a viable target for strategies to inhibit apoptosis in SCI treatment. Pkm, particularly the M2 isoform, regulates glycolysis and energy metabolism in cells. Disruptions in energy metabolism are significant in SCI, affecting neural recovery (Ren et al., 2024). Our results demonstrate differential expression of *Pkm*, reflecting alterations in energy metabolism following SCI. This aligns with a previous report highlighting its role in regulating glycolysis during cellular stress (Ren et al., 2024). This consistency shows the importance of restoring energy homeostasis for SCI rehabilitation. Cxcr4, a chemokine receptor mediating immune cell migration and inflammatory responses, is critical for SCI repair and regeneration (Kammerer et al., 2020). Its upregulation may indicate enhanced inflammatory responses and immune cell infiltration, which are essential for debris clearance and tissue remodeling post-injury. Although the role of Cxcr4 in SCI is complex, our findings support its established functions in coordinating immune responses, emphasizing its potential as a regulatory node in SCI healing (Kammerer et al., 2020). Hexa, encoding β-galactosidase A, is essential for lysosomal function and cellular metabolism. In lysosomal storage diseases, *Hexa* deficiency leads to neuronal degeneration (Cai et al., 2020). In SCI, altered expression of *Hexa* could disrupt lysosomal homeostasis, contributing to neuronal damage. After validating the key genes through bioinformatics analysis, we observed that while most genes exhibited the expected high expression, *Pkm* was computationally predicted to have low expression but displayed high expression in animal experiments. This discrepancy might arise from differences in experimental conditions, biological variability, or compensatory mechanisms within the SCI microenvironment not fully captured by the bioinformatics datasets. Casp6, Pkm, Cxcr4, and Hexa were identified as potential biomarkers due to their differential expression in hypoxic SCI. However, their clinical relevance hinges on their detectability in accessible biological samples. These biomarkers could be detected in cerebrospinal fluid or plasma in clinical settings, enabling non-invasive diagnosis and monitoring. For instance, Cxcr4 is measurable in both cerebrospinal fluid and plasma (Sojane et al., 2018), while Hexa activity can be assessed in plasma (Osher et al., 2015). Although further validation is required to detect Casp6 and Pkm in these fluids, their roles in apoptosis and glycolysis suggest potential applications in assessing injury severity and predicting prognosis (Lee et al., 2015). These biomarkers may support personalized therapeutic strategies for SCI patients by providing insights into disease progression and treatment responses.

To develop new diagnostic tools for SCI based on the identified four key genes (*Casp6*, *Pkm*, *Cxcr4*, *Hexa*), a nomogram was constructed to visually represent their contributions to SCI diagnosis, providing clinicians with a rapid diagnostic tool. The diagnostic performance of a logistic regression model based on these genes was evaluated using ROC curve analysis and AUC values to determine its clinical diagnostic efficacy. Furthermore, multivariate regression analysis was performed to clarify the independent roles of these genes in SCI diagnosis, thereby enhancing the reliability of the model. Future studies involving large-scale clinical samples are warranted to further validate the diagnostic utility of these genes and confirm their potential as reliable biomarkers for SCI.

GSEA and GSVA revealed that the high-risk group was significantly enriched in several key pathways, such as the Wang Nfkb Targets and Reactome Cd28 Dependent Pi3k Akt Signaling. The differences in pathway enrichment suggest potential molecular distinctions between the high-risk and low-risk groups and may reveal potential therapeutic targets. For example, the NF-κB signaling pathway, known for regulating inflammation and apoptosis, is often overactivated in secondary injury following SCI. Targeting this pathway may offer a new therapeutic approach for SCI (Liu et al., 2021). Similarly, the PI3K/Akt signaling pathway, crucial for various physiological functions, has been implicated in numerous diseases and may become a target for therapeutic interventions (Xu et al., 2021).

The ssGSEA algorithm was employed in this study to calculate the Hs for all samples. This method significantly enhances scoring accuracy by assessing the expression of specific gene sets in each sample. Unlike traditional gene expression scoring methods, ssGSEA does not depend on predefined classification thresholds and offers continuous scoring based on the degree of gene set enrichment. This approach mitigates subjectivity associated with threshold selection and improves the objectivity and reliability of the results (Xiao et al., 2020). In addition, single-sample Gene Set Enrichment Analysis (ssGSEA) effectively captures nonlinear variations in gene expression, providing valuable insights into complex BPs. Implementing the ssGSEA-based hypoxia scoring system in clinical practice involves several stages of validation and optimization. Initially, the system’s stability and accuracy should be verified using a larger, independent sample set to evaluate its effectiveness across different populations. Subsequent refinement of the scoring system should integrate clinical information to enhance its predictive capability. The refined system could then be utilized to develop personalized treatment plans, enabling earlier interventions for high-risk patients to improve their prognosis. Additionally, it is a valuable tool for assessing treatment efficacy and facilitating necessary adjustments to treatment strategies.

This study systematically integrated and calibrated two independent datasets, identified 186 HRDEGs through comprehensive bioinformatics analyses, and developed a robust diagnostic model for predicting SCI. These findings provide valuable insights into the mechanisms underlying SCI and the development of novel therapeutic strategies.

### 4.1 Limitations of the study

This study has several limitations. First, although two independent datasets were integrated and calibrated, the sample size might still be relatively small, potentially limiting the statistical power and generalizability of the results. Second, the diagnostic model developed for predicting SCI requires further validation in more extensive and diverse cohorts to ensure its accuracy and reliability. While our diagnostic model shows promising performance in identifying key biomarkers for SCI, its clinical applicability requires further investigation. Currently, the model is based on bioinformatics data from relatively small datasets and necessitates validation in larger and more diverse cohorts to confirm its robustness and reliability across different populations. Additionally, its effectiveness in real-world clinical scenarios may be influenced by factors such as inter-individual variability, comorbidities, and treatment interventions, which were not accounted for in this study. Therefore, future research should incorporate comprehensive clinical information and longitudinal follow-up data to refine the model and enhance its predictive accuracy. Additionally, the comprehensive bioinformatics analyses identified 186 HRDEGs, but the functional validation of these genes in the context of SCI is lacking. Future studies should address these limitations to provide more conclusive and widely applicable findings.

## Data Availability

Publicly available datasets were analyzed in this study. This data can be found at: the R package GEOquery (version 2.70.0) was used to retrieve SCI datasets GSE5296 and GSE47681 from the Gene Expression Omnibus (GEO) database (https://www.ncbi.nlm.nih.gov/geo/). Hypoxia-related genes (HRGs) were obtained from the GeneCards database (https://www.genecards.org/). Data acquisition means are specified in the methodology section under Data Source.

## Footnotes

^1^ https://www.ncbi.nlm.nih.gov/geo/

^2^ https://www.genecards.org/

^3^ https://pubmed.ncbi.nlm.nih.gov/

^4^ http://rna.sysu.edu.cn/chipbase/

^5^ http://www.microrna.gr/tarbase

^6^ https://starbase.sysu.edu.cn/

^7^ https://ctdbase.org/

^8^ https://github.com/rcastelo/GSVA
