# Supplementary material for: Exploring hypoxia-related genes in spinal cord injury: a pathway to new therapeutic targets
Source: Front Mol Neurosci. 2025 May 20;18:1565430. doi: 10.3389/fnmol.2025.1565430 (PMC12130011; doi:10.3389/fnmol.2025.1565430)
Supplement: Supplementary file 1 [file Data_Sheet_1.docx]

### Table S1 GEO Microarray Chip Information

|  | GSE5296 | GSE47681 |
| --- | --- | --- |
| Platform | GPL1261 | GPL1261 |
| Species | Mus musculus | Mus musculus |
| Tissue | Randall’s Plaque Tissues | Randall's Plaque Tissue |
| Samples in the SCI group | 6 | 8 |
| Samples in the Control group | 4 | 4 |
| Reference | / | PMID:23884949 |

GEO, Gene Expression Omnibus; SCI, Spinal Cord Injury.

**Table S2. Hypoxia-relate genes.**

| gene_symbol |
| --- |
| Ace |
| Ackr3 |
| Adm |
| Adora2b |
| Ak4 |
| Akap12 |
| Akt1 |
| Aldoa |
| Aldob |
| Aldoc |
| Alkbh5 |
| Ampd3 |
| Angpt2 |
| Angptl4 |
| Ankzf1 |
| Anxa2 |
| Arnt |
| Arnt2 |
| Atf3 |
| Atp7a |
| B3galt6 |
| B4galnt2 |
| Bax |
| Bcan |
| Bcl2 |
| Bdnf |
| Bgn |
| Bhlhe40 |
| Bnip3 |
| Bnip3l |
| Gm10480 |
| Brs3 |
| Btg1 |
| Car12 |
| Car9 |
| Camk4 |
| Casp3 |
| Casp6 |
| Casp9 |
| Cav1 |
| Ccl12 |
| Ccng2 |
| Cdkn1a |
| Cdkn1b |
| Chst2 |
| Chst3 |
| Cited2 |
| Col1a1 |
| Col5a1 |
| Cp |
| Creb1 |
| Crebbp |
| Crp |
| Csrp2 |
| Ctnnb1 |
| Cul2 |
| Cxcl12 |
| Cxcr4 |
| Dcn |
| Ddit3 |
| Ddit4 |
| Dpysl4 |
| Dtna |
| Dusp1 |
| Edn1 |
| Edn2 |
| Efna1 |
| Efna3 |
| Egfr |
| Egln1 |
| Egln2 |
| Egln3 |
| Egr1 |
| Eng |
| Gm5506 |
| Eno1 |
| Eno2 |
| Eno3 |
| Ep300 |
| Epas1 |
| Epo |
| Epor |
| Errfi1 |
| Ets1 |
| Ext1 |
| F3 |
| Fam162a |
| Fbp1 |
| Fgf2 |
| Flt1 |
| Fos |
| Fosl2 |
| Foxo3 |
| Gaa |
| Galk1 |
| Gapdh |
| Gm20899 |
| Gapdhs |
| Gbe1 |
| Gck |
| Gcnt2 |
| Glrx |
| Gpc1 |
| Gpc3 |
| Gpc4 |
| Gpi1 |
| Grhpr |
| Gys1 |
| Has1 |
| Hdlbp |
| Hexa |
| Hif1an |
| Hif3a |
| Higd1a |
| Higd1b |
| Higd1c |
| Higd2a |
| Hilpda |
| Hipk2 |
| Hk1 |
| Hk2 |
| Hmox1 |
| Hoxb9 |
| Hs3st1 |
| Hsp90aa1 |
| Hspa5 |
| Hyou1 |
| Ids |
| Ier3 |
| Igf1 |
| Igfbp1 |
| Igfbp3 |
| Il1b |
| Il6 |
| Ilvbl |
| Inha |
| Ins2 |
| Irs2 |
| Isg20 |
| Jmjd6 |
| Jun |
| Kdelr3 |
| Kdm3a |
| Kdr |
| Kif5a |
| Klf6 |
| Klf7 |
| Klhl24 |
| Lalba |
| Ldha |
| Ldhc |
| Lep |
| Lox |
| Lrp5 |
| Lxn |
| Maff |
| Map3k1 |
| Mapk1 |
| Mapk14 |
| Mapk8 |
| Mdm2 |
| Mgarp |
| Mif |
| Mmp2 |
| Mmp9 |
| Mtor |
| Mxi1 |
| Myc |
| Myh9 |
| Nagk |
| Ncan |
| Ndp |
| Ndrg1 |
| Ndst1 |
| Ndst2 |
| Nedd4l |
| Nfe2l2 |
| Nfil3 |
| Nfkb1 |
| Nos1 |
| Nos2 |
| Nos3 |
| Notch1 |
| Nr3c1 |
| P4ha1 |
| P4ha2 |
| P4htm |
| Pam |
| Pck1 |
| Pdgfb |
| Pdk1 |
| Pdk3 |
| Pfkfb3 |
| Pfkfb4 |
| Pfkl |
| Pfkp |
| Pgam2 |
| Pgf |
| Pgk1 |
| Pgm2 |
| Pgm1 |
| Phkg1 |
| Pik3cg |
| Pim1 |
| Pklr |
| Pkm |
| Pkp1 |
| Plac8 |
| Plaur |
| Plin2 |
| Pnrc1 |
| Ppargc1a |
| Ppp1r15a |
| Ppp1r3c |
| Prdx5 |
| Prkaa2 |
| Prkca |
| Psma7 |
| Pten |
| Ptgs2 |
| Pygm |
| Rbpj |
| Rest |
| Rhoa |
| Rora |
| Rragd |
| Rwdd3 |
| S100a4 |
| Sap30 |
| Scarb1 |
| Sdc2 |
| Sdc3 |
| Sdc4 |
| Sdhb |
| Selenbp1 |
| Selenbp2 |
| Serpine1 |
| Sesn2 |
| Setd2 |
| Siah2 |
| Sirt1 |
| Slc2a1 |
| Slc2a3 |
| Slc2a5 |
| Slc37a4 |
| Slc6a6 |
| Sod1 |
| Sp1 |
| Spp1 |
| Src |
| Srpx |
| Stat3 |
| Stbd1 |
| Stc1 |
| Stc2 |
| Sult2b1 |
| Tek |
| Tes |
| Tgfb1 |
| Tgfb3 |
| Tgfbi |
| Tgm2 |
| Th |
| Thbs1 |
| Tiparp |
| Tktl1 |
| Tlr4 |
| Tmem45a |
| Tnf |
| Tnfaip3 |
| Trp53 |
| Tpbg |
| Tpd52 |
| Tpi1 |
| Tpst2 |
| Ugp2 |
| Vegfa |
| Vegfc |
| Vhl |
| Vldlr |
| Wsb1 |
| Xdh |
| Xpnpep1 |
| Zfp36 |
| Zfp292 |

Table S3. Nucleotide sequences of qPCR primers

| Primer name | Primer information | Base sequence (5`-3`) | | Tm Value | CG% | Product length |
| --- | --- | --- | --- | --- | --- | --- |
| M-ACTIN | NM_007393.5 | F | CTGAGAGGGAAATCGTGCGT | 60 | 55 | 208 |
|  |  | R | CCACAGGATTCCATACCCAAGA | 61.3 | 50 |  |
| M-Caspase-6 | NM_009811 | F | AGCCGAGCAGTACAAGATGGA | 59.9 | 52.4 | 194 |
|  |  | R | GCAGGAGTTCTTCTGCTCTGAG | 58.5 | 54.5 |  |
| M-Pkm | NM_001253883 | F | ACCTGAGATCCGGACTGGACT | 61.9 | 57.1 | 114 |
|  |  | R | TCGTCACACTTCTCCATGTAAGC | 60.6 | 47 |  |
| M-Cxcr4 | NM_009911.3 | F | GGAGCATGACGGACAAGTACC | 59.3 | 57.1 | 167 |
|  |  | R | GAGAACGCTGCTGTAGAGGTTG | 59.4 | 54.5 |  |
| M-Hexa | NM_010421 | F | GATACACTGGATGTCATGGCATAC | 58.7 | 45.8 | 275 |
|  |  | R | GAGTAGCAAGGTGTTAATAACCCAG | 59.1 | 44 |  |

### Table S4. Results of GO and KEGG Enrichment Analysis for HRDEGs.

| Ontology | ID | Description | GeneRatio | BgRatio | p-value | p.adjust |
| --- | --- | --- | --- | --- | --- | --- |
| BP | GO:0001666 | response to Hypoxia | 33/185 | 219/28943 | 1.12962E-35 | 4.7715E-32 |
| BP | GO:0036293 | response to decreSCIed oxygen levels | 33/185 | 246/28943 | 6.10572E-34 | 1.28953E-30 |
| BP | GO:0033002 | muscle cell proliferation | 32/185 | 266/28943 | 2.28253E-31 | 3.21381E-28 |
| BP | GO:0016052 | carbohydrate catabolic process | 27/185 | 154/28943 | 3.74059E-31 | 3.95006E-28 |
| BP | GO:0070482 | response to oxygen levels | 33/185 | 305/28943 | 8.52788E-31 | 7.20435E-28 |
| CC | GO:0062023 | collagen-containing extracellular matrix | 22/185 | 396/28804 | 1.45618E-14 | 4.61611E-12 |
| CC | GO:0045121 | membrane raft | 20/185 | 394/28804 | 1.29719E-12 | 1.43586E-10 |
| CC | GO:0098857 | membrane microdomain | 20/185 | 395/28804 | 1.35885E-12 | 1.43586E-10 |
| CC | GO:0090575 | RNA polymerSCIe II transcription regulator complex | 16/185 | 242/28804 | 4.9107E-12 | 3.89173E-10 |
| CC | GO:0005901 | caveola | 10/185 | 96/28804 | 6.54462E-10 | 4.14929E-08 |
| MF | GO:0061629 | RNA polymerSCIe II-specific DNA-binding transcription factor binding | 20/185 | 376/28404 | 7.06346E-13 | 2.14404E-10 |
| MF | GO:0048029 | monosaccharide binding | 12/185 | 91/28404 | 8.199E-13 | 2.14404E-10 |
| MF | GO:0030246 | carbohydrate binding | 17/185 | 277/28404 | 4.23348E-12 | 7.38037E-10 |
| MF | GO:0019200 | carbohydrate kinSCIe activity | 6/185 | 21/28404 | 3.52092E-09 | 4.6036E-07 |
| MF | GO:0019838 | growth factor binding | 11/185 | 152/28404 | 5.18084E-09 | 5.41915E-07 |
| KEGG | mmu04066 | HIF-1 signaling pathway - Mus musculus (house mouse) | 28/148 | 116/9722 | 1.81499E-26 | 4.70083E-24 |
| KEGG | mmu05205 | Proteoglycans in cancer - Mus musculus (house mouse) | 28/148 | 204/9722 | 2.28022E-19 | 2.95289E-17 |
| KEGG | mmu05418 | Fluid shear stress and atherosclerosis - Mus musculus (house mouse) | 24/148 | 148/9722 | 2.01671E-18 | 1.74109E-16 |
| KEGG | mmu04933 | AGE-RAGE signaling pathway in diabetic complications - Mus musculus (house mouse) | 19/148 | 101/9722 | 5.12279E-16 | 3.317E-14 |
| KEGG | mmu05210 | Colorectal cancer - Mus musculus (house mouse) | 15/148 | 88/9722 | 3.26997E-12 | 1.69385E-10 |

GO: Gene Ontology. BP: Biological Process. CC: Cellular Component. MF: Molecular Function. KEGG: Kyoto Encyclopedia of Genes and Genomes. HRDEGs: Hypoxia-Related Differentially Expressed Genes.

### Table S5. Results of GSEA for Combined Datasets.

| ID | setSize | enrichmentScore | NES | p-value | p.adjust | q-value |
| --- | --- | --- | --- | --- | --- | --- |
| BIOCARTA_NFKB_PATHWAY | 21 | 0.761804532 | 1.87546042 | 0.000313629 | 0.001336445 | 0.000694633 |
| YAUCH_HEDGEHOG_SIGNALING_PARACRINE_DN | 267 | -0.355781151 | -1.579937685 | 4.75E-05 | 0.000257889 | 0.000134041 |
| BIOCARTA_TGFB_PATHWAY | 17 | 0.645102766 | 1.530800636 | 0.043402778 | 0.087729913 | 0.045598679 |
| ONGUSAHA_TP53_TARGETS | 39 | 0.675712778 | 1.898410718 | 0.000381912 | 0.00159168 | 0.000827295 |

GSEA: Gene Set Enrichment Analysis.

### Table S6. Results of GSVA for Combined Datasets.

|  | logFC | AveExpr | t | P.Value |
| --- | --- | --- | --- | --- |
| BIOCARTA_NFKB_PATHWAY | 1.56190096 | 0.084880365 | 20.19278477 | 4.91807E-18 |
| BIOCARTA_RELA_PATHWAY | 1.504643607 | 0.085299458 | 18.94362755 | 2.54376E-17 |
| HESS_TARGETS_OF_HOXA9_AND_MEIS1_DN | 1.471139811 | 0.073162474 | 14.34195898 | 2.7097E-14 |
| HEVNER_TELENCEPHALON_MICROGLIA | 1.852605706 | 0.091646792 | 14.38322102 | 2.52633E-14 |
| HEVNER_TELENCEPHALON_VSCICULAR_ENDOTHELIUM_AND_MENINGEAL_CELLS | 1.501348299 | 0.049713121 | 8.049642382 | 1.03833E-08 |
| ICHIBA_GRAFT_VERSUS_HOST_DISESCIE_D7_UP | 1.46492619 | 0.084872631 | 16.47739068 | 8.74241E-16 |
| LIAN_LIPA_TARGETS_3M | 1.457533152 | 0.081173911 | 15.93125601 | 2.03016E-15 |
| REACTOME_CSCIPSCIE_ACTIVATION_VIA_DEATH_RECEPTORS_IN_THE_PRESENCE_OF_LIGAND | 1.546266058 | 0.080786402 | 11.83501262 | 2.62037E-12 |
| REACTOME_GABA_SYNTHESIS_RELESCIE_REUPTAKE_AND_DEGRADATION | -1.482934662 | -0.069418301 | -9.879114055 | 1.50874E-10 |
| REACTOME_LAMININ_INTERACTIONS | 1.544727232 | 0.0836609 | 9.560518152 | 3.05448E-10 |
| REACTOME_PRESYNAPTIC_DEPOLARIZATION_AND_CALCIUM_CHANNEL_OPENING | -1.514801629 | -0.070334265 | -9.830022912 | 1.68052E-10 |
| REACTOME_REGULATION_BY_C_FLIP | 1.478255198 | 0.070483272 | 12.13805215 | 1.45595E-12 |
| REACTOME_SYNDECAN_INTERACTIONS | 1.496419202 | 0.100655783 | 15.87891684 | 2.2036E-15 |
| RUAN_RESPONSE_TO_TNF_TROGLITAZONE_UP | 1.458192876 | 0.100271607 | 15.83279231 | 2.3691E-15 |
| RUAN_RESPONSE_TO_TNF_UP | 1.56654005 | 0.107840231 | 16.54583637 | 7.87911E-16 |
| STEARMAN_TUMOR_FIELD_EFFECT_UP | 1.472904479 | 0.109174953 | 10.78958181 | 2.15825E-11 |
| STEGER_ADIPOGENESIS_DN | 1.484620612 | 0.061500705 | 13.55296394 | 1.06686E-13 |
| WP_CHOLESTEROL_BIOSYNTHESIS | -1.66607049 | -0.084374405 | -9.144106061 | 7.83365E-10 |
| WP_MACROPHAGE_MARKERS | 1.657497113 | 0.080788632 | 12.06900372 | 1.66305E-12 |
| WP_TYROBP_CAUSAL_NETWORK_IN_MICROGLIA | 1.651700773 | 0.095539966 | 19.088625 | 2.09205E-17 |

**Table S7. Logistic regression**

|  | Gene | OR | OR_1 | OR_2 | pvalue | OR(95% CI) |
| --- | --- | --- | --- | --- | --- | --- |
| 1 | Tgfbi | -2.456985385 | -5.77628289 | -0.975589361 | 0.022 | -2.46(-5.78~-0.98) |
| 3 | Hdlbp | -15.73234489 | -33.06919074 | -5.583146137 | 0.018 | -15.73(-33.07~-5.58) |
| 4 | Casp6 | -8.321421423 | -20.09493278 | -3.511802456 | 0.021 | -8.32(-20.09~-3.51) |
| 5 | Scarb1 | -6.693885418 | -15.66690108 | -2.303963533 | 0.032 | -6.69(-15.67~-2.3) |
| 6 | Hspa5 | -13.63046919 | -31.06089358 | -4.776936325 | 0.031 | -13.63(-31.06~-4.78) |
| 7 | Pfkp | 23.71766365 | 9.032111927 | 56.82516559 | 0.033 | 23.72(9.03~56.83) |
| 9 | Lox | -1.682727336 | -3.385701531 | -0.64505579 | 0.011 | -1.68(-3.39~-0.65) |
| 10 | Mmp2 | -4.638181618 | -9.748640409 | -1.802823599 | 0.016 | -4.64(-9.75~-1.8) |
| 12 | Bgn | -5.21401306 | -12.58252762 | -1.835281322 | 0.047 | -5.21(-12.58~-1.84) |
| 13 | Higd1a | 20.84946166 | 7.921447124 | 56.92943139 | 0.04 | 20.85(7.92~56.93) |
| 14 | Lxn | -5.561069599 | -10.91319334 | -2.397964571 | 0.0057 | -5.56(-10.91~-2.4) |
| 17 | Bcan | 9.890060116 | 2.627817256 | 22.74423766 | 0.047 | 9.89(2.63~22.74) |
| 22 | Slc37a4 | 13.97969777 | 4.973739412 | 29.08709023 | 0.015 | 13.98(4.97~29.09) |
| 24 | Angptl4 | -5.904368438 | -13.45864777 | -2.270199401 | 0.025 | -5.9(-13.46~-2.27) |
| 25 | Galk1 | -10.82443804 | -22.37288532 | -4.484357136 | 0.012 | -10.82(-22.37~-4.48) |
| 26 | Ptgs2 | -1.770405564 | -3.7795765 | -0.491621308 | 0.026 | -1.77(-3.78~-0.49) |
| 28 | Pkm | 34.75051496 | 13.8923968 | 80.79494008 | 0.023 | 34.75(13.89~80.79) |
| 29 | Sult2b1 | 3.856869214 | 1.141028789 | 8.006591374 | 0.022 | 3.86(1.14~8.01) |
| 31 | F3 | -6.035438372 | -12.4802478 | -2.360960332 | 0.011 | -6.04(-12.48~-2.36) |
| 34 | Cp | -5.179910174 | -12.1630151 | -1.835494981 | 0.033 | -5.18(-12.16~-1.84) |
| 35 | Tgm2 | -2.941586839 | -6.410314599 | -1.187291227 | 0.014 | -2.94(-6.41~-1.19) |
| 36 | Ddit3 | -9.434897408 | -23.26317017 | -3.575274444 | 0.034 | -9.43(-23.26~-3.58) |
| 37 | Cxcl12 | -5.723834009 | -12.37363027 | -1.785244856 | 0.028 | -5.72(-12.37~-1.79) |
| 38 | Selenbp1 | -6.665801433 | -14.24836863 | -1.647103867 | 0.031 | -6.67(-14.25~-1.65) |
| 40 | Ackr3 | -4.779074751 | -9.933849077 | -1.655059013 | 0.016 | -4.78(-9.93~-1.66) |
| 41 | Sdc4 | -8.639125528 | -20.89189177 | -3.389917359 | 0.028 | -8.64(-20.89~-3.39) |
| 43 | Ext1 | -16.51480036 | -37.75264453 | -6.244807787 | 0.024 | -16.51(-37.75~-6.24) |
| 45 | Vldlr | 10.51692388 | 4.53847078 | 22.06866436 | 0.0088 | 10.52(4.54~22.07) |
| 46 | Ndst2 | -6.98880041 | -15.80306282 | -1.878080703 | 0.033 | -6.99(-15.8~-1.88) |
| 47 | Higd2a | 13.10366196 | 2.8311113 | 30.35871584 | 0.048 | 13.1(2.83~30.36) |
| 48 | Sdhb | 20.08823176 | 5.96362122 | 44.34446338 | 0.029 | 20.09(5.96~44.34) |
| 49 | Bhlhe40 | -11.83425244 | -26.30002075 | -4.787181646 | 0.018 | -11.83(-26.3~-4.79) |
| 52 | Sp1 | -16.51514392 | -35.3909267 | -6.18064237 | 0.017 | -16.52(-35.39~-6.18) |
| 54 | Dpysl4 | 14.03316512 | 4.744019652 | 32.3601622 | 0.037 | 14.03(4.74~32.36) |
| 55 | Kdelr3 | -3.826190156 | -8.834266796 | -1.523656907 | 0.02 | -3.83(-8.83~-1.52) |
| 56 | Notch1 | -6.045776096 | -12.73420926 | -2.042435415 | 0.02 | -6.05(-12.73~-2.04) |
| 59 | Nfil3 | -6.241758717 | -13.42772389 | -2.363103944 | 0.018 | -6.24(-13.43~-2.36) |
| 60 | Maff | -3.604249587 | -7.531523952 | -1.208340647 | 0.018 | -3.6(-7.53~-1.21) |
| 61 | Anxa2 | -6.22462563 | -15.12581919 | -2.425015269 | 0.032 | -6.22(-15.13~-2.43) |
| 62 | Serpine1 | -1.357772581 | -2.969515988 | -0.504702055 | 0.017 | -1.36(-2.97~-0.5) |
| 63 | Dtna | 9.214733849 | 3.012642747 | 18.95807644 | 0.018 | 9.21(3.01~18.96) |
| 65 | Klf7 | 6.11999869 | 1.358427201 | 13.53532672 | 0.039 | 6.12(1.36~13.54) |
| 68 | Nagk | -10.78056488 | -24.11211883 | -4.302731695 | 0.021 | -10.78(-24.11~-4.3) |
| 69 | Igf1 | -2.411669144 | -5.355893428 | -0.894144625 | 0.024 | -2.41(-5.36~-0.89) |
| 70 | Mapk1 | 17.55140497 | 4.744618654 | 40.60215372 | 0.039 | 17.55(4.74~40.6) |
| 72 | Zfp292 | 11.14703784 | 3.813591604 | 23.38179403 | 0.019 | 11.15(3.81~23.38) |
| 73 | Tnf | -2.914314665 | -6.448413915 | -0.47361493 | 0.045 | -2.91(-6.45~-0.47) |
| 74 | Ier3 | -2.673634363 | -5.62211995 | -0.820243225 | 0.023 | -2.67(-5.62~-0.82) |
| 75 | Ldha | -5.921543334 | -15.2049797 | -2.313163144 | 0.036 | -5.92(-15.2~-2.31) |
| 80 | Rora | 7.695794559 | 2.928267687 | 14.83881715 | 0.0079 | 7.7(2.93~14.84) |
| 82 | Arnt2 | 15.04427658 | 5.521043149 | 29.691341 | 0.01 | 15.04(5.52~29.69) |
| 83 | Csrp2 | -6.139943891 | -13.1169459 | -2.417927113 | 0.015 | -6.14(-13.12~-2.42) |
| 84 | Rwdd3 | 4.937085934 | 1.558491008 | 9.884108681 | 0.015 | 4.94(1.56~9.88) |
| 85 | Ctnnb1 | -16.21888551 | -32.32548821 | -6.477411046 | 0.0094 | -16.22(-32.33~-6.48) |
| 86 | Sdc3 | -12.88824181 | -28.83206787 | -4.551430028 | 0.023 | -12.89(-28.83~-4.55) |
| 87 | Hk1 | 25.71330395 | 8.808298824 | 56.78183079 | 0.028 | 25.71(8.81~56.78) |
| 88 | Vegfa | 12.75535439 | 5.201253865 | 28.52164239 | 0.017 | 12.76(5.2~28.52) |
| 89 | Mapk8 | 10.97100846 | 4.479993325 | 29.19914781 | 0.033 | 10.97(4.48~29.2) |
| 91 | Hilpda | -2.869399377 | -6.113735758 | -0.525844598 | 0.039 | -2.87(-6.11~-0.53) |
| 92 | Gpc4 | -8.693234226 | -18.07456228 | -3.32928952 | 0.015 | -8.69(-18.07~-3.33) |
| 93 | Ids | 28.3795397 | 10.17358172 | 72.79016302 | 0.046 | 28.38(10.17~72.79) |
| 96 | Cdkn1a | -3.383218401 | -7.486840886 | -0.993804167 | 0.03 | -3.38(-7.49~-0.99) |
| 97 | Ak4 | 7.526899495 | 2.763276451 | 14.87758567 | 0.011 | 7.53(2.76~14.88) |
| 101 | Ets1 | -8.64600183 | -18.63917301 | -3.169122077 | 0.023 | -8.65(-18.64~-3.17) |
| 102 | Ndst1 | -8.365484987 | -17.01260861 | -2.587938686 | 0.018 | -8.37(-17.01~-2.59) |
| 103 | Bdnf | 5.613354547 | 1.07988345 | 12.83303226 | 0.049 | 5.61(1.08~12.83) |
| 104 | Xpnpep1 | -11.5724289 | -25.31741067 | -4.40227298 | 0.02 | -11.57(-25.32~-4.4) |
| 107 | Tmem45a | -3.607965401 | -8.308518159 | -1.035918511 | 0.045 | -3.61(-8.31~-1.04) |
| 113 | Fosl2 | -6.711095971 | -14.957756 | -2.72401496 | 0.015 | -6.71(-14.96~-2.72) |
| 114 | Bcl2 | 9.612380398 | 2.391099384 | 19.64625387 | 0.023 | 9.61(2.39~19.65) |
| 120 | Ncan | -5.256463747 | -10.53046177 | -1.370181053 | 0.02 | -5.26(-10.53~-1.37) |
| 122 | Col1a1 | -1.417088584 | -3.033051332 | -0.437527662 | 0.023 | -1.42(-3.03~-0.44) |
| 123 | Pdk1 | -11.86546672 | -25.01559918 | -4.006649438 | 0.019 | -11.87(-25.02~-4.01) |
| 126 | Stat3 | -7.287771424 | -14.98132317 | -2.836845229 | 0.012 | -7.29(-14.98~-2.84) |
| 129 | Hipk2 | 10.86648465 | 3.494777678 | 22.82145994 | 0.019 | 10.87(3.49~22.82) |
| 133 | Wsb1 | -4.628553693 | -9.775585951 | -0.867105554 | 0.035 | -4.63(-9.78~-0.87) |
| 137 | Pdk3 | 16.18755831 | 5.983041029 | 36.30000228 | 0.025 | 16.19(5.98~36.3) |
| 138 | Ugp2 | -19.03531113 | -38.37826799 | -8.124321771 | 0.008 | -19.04(-38.38~-8.12) |
| 139 | P4ha1 | -4.06694359 | -8.803972791 | -0.973020924 | 0.034 | -4.07(-8.8~-0.97) |
| 142 | Hsp90aa1 | 7.493346291 | 2.058471001 | 15.57898903 | 0.023 | 7.49(2.06~15.58) |
| 143 | Tiparp | -15.27712621 | -38.05270272 | -5.203368047 | 0.049 | -15.28(-38.05~-5.2) |
| 145 | Ace | 12.18327305 | 3.915498917 | 25.1653234 | 0.019 | 12.18(3.92~25.17) |
| 151 | Hif1an | 15.90749713 | 6.004231875 | 33.31892932 | 0.016 | 15.91(6~33.32) |
| 152 | Prkaa2 | 10.2720032 | 4.097564133 | 30.90613562 | 0.047 | 10.27(4.1~30.91) |
| 153 | Rragd | 17.08586204 | 5.895377633 | 37.62883216 | 0.024 | 17.09(5.9~37.63) |
| 155 | Pnrc1 | 25.10659139 | 6.777911219 | 58.40419029 | 0.044 | 25.11(6.78~58.4) |
| 162 | Dcn | -5.70838674 | -12.4492838 | -2.226709754 | 0.018 | -5.71(-12.45~-2.23) |
| 163 | Siah2 | 14.81599473 | 6.12644278 | 34.03740287 | 0.018 | 14.82(6.13~34.04) |
| 164 | Hmox1 | -1.106376262 | -2.733860213 | -0.434720786 | 0.024 | -1.11(-2.73~-0.43) |
| 167 | Cxcr4 | -2.851132053 | -6.739065836 | -1.240612271 | 0.017 | -2.85(-6.74~-1.24) |
| 168 | Dusp1 | -12.39864684 | -29.5518973 | -3.984060448 | 0.045 | -12.4(-29.55~-3.98) |
| 169 | Angpt2 | -4.159553176 | -9.738715067 | -1.007009999 | 0.046 | -4.16(-9.74~-1.01) |
| 170 | Hexa | -3.509507996 | -7.773850128 | -1.562030586 | 0.011 | -3.51(-7.77~-1.56) |
| 171 | Cav1 | -6.936521773 | -16.72448743 | -2.550064019 | 0.037 | -6.94(-16.72~-2.55) |
| 174 | Kdr | -3.004754415 | -6.740924984 | -0.782922205 | 0.038 | -3(-6.74~-0.78) |
| 175 | Fgf2 | -3.738983253 | -8.739531437 | -0.940889366 | 0.042 | -3.74(-8.74~-0.94) |
| 177 | Pdgfb | -14.37271666 | -33.51188979 | -5.130652306 | 0.033 | -14.37(-33.51~-5.13) |
| 180 | Plac8 | -1.373277098 | -2.836459287 | -0.40924497 | 0.02 | -1.37(-2.84~-0.41) |
| 181 | Hoxb9 | 9.99672637 | 4.163788146 | 23.13840875 | 0.017 | 10(4.16~23.14) |

**Table S8. mRNA - TF interaction network nodes.**

| mRNA | TF |
| --- | --- |
| Casp6 | PHF8 |
| Cxcr4 | ATF3 |
| Cxcr4 | BCL11B |
| Cxcr4 | BRD4 |
| Cxcr4 | CBFB |
| Cxcr4 | CEBPA |
| Cxcr4 | CEBPB |
| Cxcr4 | EP300 |
| Cxcr4 | ESR2 |
| Cxcr4 | ESRRB |
| Cxcr4 | ETS1 |
| Cxcr4 | EZH2 |
| Cxcr4 | FLI1 |
| Cxcr4 | FOXA2 |
| Cxcr4 | FOXH1 |
| Cxcr4 | FOXP3 |
| Cxcr4 | HDAC1 |
| Cxcr4 | HDAC3 |
| Cxcr4 | IKZF1 |
| Cxcr4 | IRF3 |
| Cxcr4 | IRF5 |
| Cxcr4 | IRF8 |
| Cxcr4 | JARID2 |
| Cxcr4 | KLF4 |
| Cxcr4 | MTF2 |
| Cxcr4 | NANOG |
| Cxcr4 | NR1D1 |
| Cxcr4 | NR3C1 |
| Cxcr4 | NRF1 |
| Cxcr4 | PAX5 |
| Cxcr4 | PCGF1 |
| Cxcr4 | PHF8 |
| Cxcr4 | POU5F1 |
| Cxcr4 | PPARA |
| Cxcr4 | PPARG |
| Cxcr4 | RNF2 |
| Cxcr4 | RUNX1 |
| Cxcr4 | RUNX2 |
| Cxcr4 | SP3 |
| Cxcr4 | SPI1 |
| Cxcr4 | STAT5 |
| Cxcr4 | SUZ12 |
| Cxcr4 | TCF3 |
| Cxcr4 | TCF7L1 |
| Cxcr4 | WDR5 |
| Cxcr4 | ZNF384 |
| Hexa | ARNTL |
| Hexa | BHLHE40 |
| Hexa | BRD4 |
| Hexa | CLOCK |
| Hexa | HDAC1 |
| Hexa | MYC |
| Hexa | PCGF1 |
| Hexa | PCGF6 |
| Hexa | RELA |
| Hexa | USF1 |
| Hexa | USF2 |
| Pkm | BHLHE40 |
| Pkm | CEBPB |
| Pkm | KLF4 |
| Pkm | MYOD1 |
| Pkm | MYOG |
| Pkm | POLR2A |
| Pkm | RXRA-B |
| Pkm | SP1 |
| Pkm | SPI1 |
| Pkm | STAT1 |

**Table S9. mRNA-miRNA interaction network nodes.**

| mRNA | miRNA |
| --- | --- |
| Hexa | mmu-miR-706 |
| Casp6 | mmu-miR-106a-5p |
| Casp6 | mmu-miR-132-3p |
| Casp6 | mmu-miR-145a-3p |
| Casp6 | mmu-miR-17-5p |
| Casp6 | mmu-miR-181a-5p |
| Casp6 | mmu-miR-181b-5p |
| Casp6 | mmu-miR-181c-5p |
| Casp6 | mmu-miR-181d-5p |
| Casp6 | mmu-miR-20b-5p |
| Casp6 | mmu-miR-24-3p |
| Casp6 | mmu-miR-30a-5p |
| Casp6 | mmu-miR-30b-5p |
| Casp6 | mmu-miR-30c-5p |
| Casp6 | mmu-miR-30d-5p |
| Casp6 | mmu-miR-30e-5p |
| Casp6 | mmu-miR-378a-5p |
| Casp6 | mmu-miR-467f |
| Casp6 | mmu-miR-669k-3p |
| Casp6 | mmu-miR-712-5p |
| Casp6 | mmu-miR-93-5p |
| Pkm | mmu-miR-122-5p |
| Pkm | mmu-miR-125a-3p |
| Pkm | mmu-miR-125b-5p |
| Pkm | mmu-miR-130b-3p |
| Pkm | mmu-miR-142a-3p |
| Pkm | mmu-miR-145a-5p |
| Pkm | mmu-miR-150-5p |
| Pkm | mmu-miR-222-3p |
| Pkm | mmu-miR-322-5p |
| Pkm | mmu-miR-378a-5p |
| Pkm | mmu-miR-497a-5p |
| Pkm | mmu-miR-706 |
| Pkm | mmu-miR-762 |
| Cxcr4 | mmu-miR-132-3p |
| Cxcr4 | mmu-miR-146a-5p |
| Cxcr4 | mmu-miR-146b-5p |
| Cxcr4 | mmu-miR-181b-5p |
| Cxcr4 | mmu-miR-181d-5p |
| Cxcr4 | mmu-miR-1a-3p |
| Cxcr4 | mmu-miR-206-3p |
| Cxcr4 | mmu-miR-21a-3p |
| Cxcr4 | mmu-miR-218-5p |
| Cxcr4 | mmu-miR-25-3p |
| Cxcr4 | mmu-miR-381-3p |
| Cxcr4 | mmu-miR-467a-5p |
| Cxcr4 | mmu-miR-467b-5p |
| Cxcr4 | mmu-miR-467d-5p |
| Cxcr4 | mmu-miR-467e-5p |
| Cxcr4 | mmu-miR-467f |
| Cxcr4 | mmu-miR-467h |
| Cxcr4 | mmu-miR-669k-3p |
| Cxcr4 | mmu-miR-9-5p |

**Table S10. MRNA - RBP interaction network nodes.**

| mRNA | RBP |
| --- | --- |
| Cxcr4 | Dazl |
| Hexa | Ctcf |
| Hexa | Elavl1 |
| Hexa | Jarid2 |
| Hexa | Lin28a |
| Hexa | Pabpc1 |
| Hexa | Srrm4 |
| Hexa | Srsf2 |
| Hexa | U2af2 |
| Hexa | Zfp36 |
| Pkm | Apc |
| Pkm | Celf1 |
| Pkm | Celf2 |
| Pkm | Cirbp |
| Pkm | Cstf2 |
| Pkm | Ctcf |
| Pkm | Ddx5 |
| Pkm | Eif3c |
| Pkm | Elavl1 |
| Pkm | Ezh2 |
| Pkm | Fip1l1 |
| Pkm | Fus |
| Pkm | Hnrnpc |
| Pkm | Hnrnpf |
| Pkm | Jarid2 |
| Pkm | Larp1 |
| Pkm | Lin28a |
| Pkm | Mbnl1 |
| Pkm | Mbnl2 |
| Pkm | Mbnl3 |
| Pkm | Msi1 |
| Pkm | Nacc1 |
| Pkm | Nxf1 |
| Pkm | Pabpc1 |
| Pkm | Peg10 |
| Pkm | Pspc1 |
| Pkm | Ptbp1 |
| Pkm | Ptbp2 |
| Pkm | Ptbp3 |
| Pkm | Rbfox1 |
| Pkm | Rbfox2 |
| Pkm | Rbfox3 |
| Pkm | Rbm10 |
| Pkm | Rbm15 |
| Pkm | Sfpq |
| Pkm | Srrm4 |
| Pkm | Srsf1 |
| Pkm | Srsf2 |
| Pkm | Srsf3 |
| Pkm | Srsf4 |
| Pkm | Srsf5 |
| Pkm | Srsf6 |
| Pkm | Srsf7 |
| Pkm | Suz12 |
| Pkm | Taf15 |
| Pkm | Tardbp |
| Pkm | Tet2 |
| Pkm | Trim71 |
| Pkm | U2af2 |
| Pkm | Ythdc2 |
| Pkm | Ythdf1 |
| Pkm | Zfp36 |
| Pkm | Zfp36l1 |
| Pkm | Zfp871 |

**Table S11. MRNA - Drug interaction network nodes.**

| mRNA | Drug |
| --- | --- |
| CD36 | Rosiglitazone |
| MMP1 | Particulate Matter |
| MMP1 | sodium arsenite |
| MMP1 | Tetrachlorodibenzodioxin |
| MMP1 | Tobacco Smoke Pollution |
| RB1 | palbociclib |
| RB1 | Resveratrol |
| RB1 | Sirolimus |
| RB1 | Tretinoin |
| TLR4 | Lipopolysaccharides |
| TNF | 1-Methyl-4-phenylpyridinium |
| TNF | bisphenol A |
| TNF | Calcimycin |
| TNF | Dinoprostone |
| TNF | Glucose |
| TNF | lipopolysaccharide, E coli O55-B5 |
| TNF | lipopolysaccharide, Escherichia coli O111 B4 |
| TNF | Lipopolysaccharides |
| TNF | Mustard Gas |
| TNF | Particulate Matter |
| TNF | Quercetin |
| TNF | Reactive Oxygen Species |
| TNF | Silicon Dioxide |
| TNF | sodium arsenite |
| TNF | Tetrachlorodibenzodioxin |
| TNF | Tetradecanoylphorbol Acetate |
| TNF | Thalidomide |
| TNF | Vehicle Emissions |
